# Off–On Photo- and Redox-Triggered Anion Transport Using an Indole-Based Hydrogen Bond Switch

**DOI:** 10.1021/acsomega.4c07880

**Published:** 2024-11-01

**Authors:** Manzoor Ahmad, Andrew Muir, Matthew J. Langton

**Affiliations:** Chemistry Research Laboratory, Mansfield Road, Oxford OX1 3TA, U.K.

## Abstract

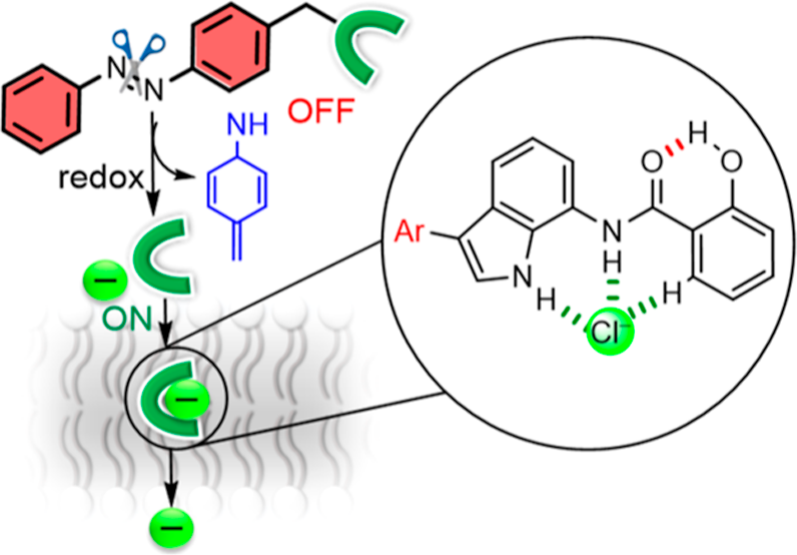

A stimulus-responsive indole-based hydrogen bonding switch is reported, which enables off–on activation of transmembrane ion transport in response to photo- and redox triggers. This is achieved by alkylation of an indole-based anionophore, preorganized through intramolecular hydrogen bonding, with *o*-nitrobenzyl and azobenzene cages. This renders the anionophore inactive through formation of a six-membered intramolecular hydrogen bonding interaction and locking of the anion binding protons. Decaging with biologically relevant light and redox stimuli leads to efficient activation of anion transport across lipid bilayer membranes by unlocking the hydrogen bond donors, such that they are now available for anion binding and transport.

## 1 Introduction

Small-molecule-based synthetic ionophores have emerged as an important class of compounds with potential applications as anticancer agents or to treat channelopathies—diseases that arise due to the malfunctioning of natural ion transport systems.^1,2^ Examples that have shown potential anticancer applications include prodigiosin,^3^ tambjamine,^4^ bis-sulfonamides,^5^ urea/thioureas,^6,7^ calix[4]pyrroles,^8^ and squaramide^9^ derivatives. Prodigiosin, a potent HCl cotransporter, remains the benchmark against which synthetic derivatives are measured and features convergent pyrrole-based hydrogen bond donors for chloride recognition. Analogous indole derivatives have also garnered increasing attention with regards to anion recognition and transport.^10−12^

In the context of exploiting anionophores as potential therapeutics, the potential for undesired cytotoxicity to healthy cells is a drawback associated with untargeted anionophores. The design of spatiotemporally targeted and activated derivatives is therefore essential. In this regard, a variety of stimulus-responsive ion transport systems have been recently developed with the aim of overcoming those side effects, triggered by diverse stimuli including light,^13−24^ membrane potential,^25^ pH,^26,27^ enzymes,^28,29^ and redox.^30,31^ The latter three stimuli are particularly relevant to targeted ionophore activation in cancer cells, which are characterized by low pH values,^32^ and overexpression of certain chemical moieties including glutathione,^33^ esterases,^34^ H_2_O_2_,^35^ and H_2_S.^36,37^

Recently, we utilized dynamic hydrogen bonding interactions of 2,6-dihydroxyisophthalamide-based anionophores to generate responsive ion transport systems that can be activated using the chemical signatures of cancer cells, namely, esterase, H_2_O_2_, or H_2_S.^38^ In addition to these signatures, low oxygen (i.e., hypoxia)-induced overexpression of reductase enzymes is a hallmark of solid tumors.^39,40^ This feature has been utilized for the activation of azobenzene-based prodrugs in which reduction of an azo moiety triggers prodrug activation.^41−43^ We envisaged that such a concept could be utilized to activate an indole-based anionophore by attaching an azobenzene handle to a suitably designed indole-based hydrogen bond switch to render it inactive. Subsequent reduction of this would lead to the activation of ion transport by changing the state of the switch.

**Figure 1:**
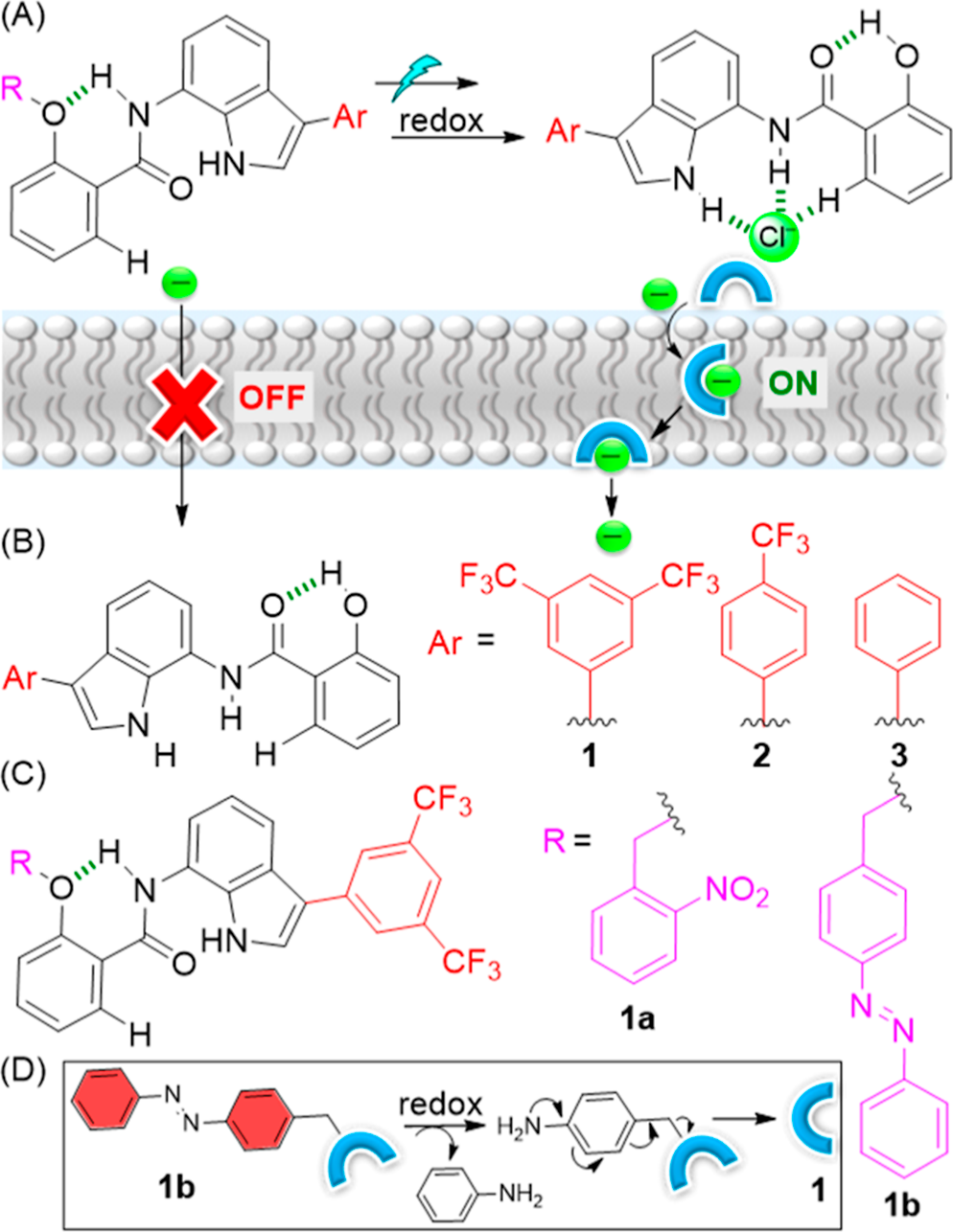
(A) Schematic representation of stimulus-responsive anion transport. Chemical structures of (B) active transporters **1–3** and (C) caged protransporters **1a** and **1b**. (D) Illustration of redox-triggered anionophore activation.

Herein, we demonstrate this concept using hydroxyindole amide-based transporters (Figure 1A). Alkylation of the free hydroxyl group with *ortho*-nitrobenzyl (ONB)- and azobenzene-based stimulus-responsive cages renders the transporter inactive by locking the amide bond through intramolecular hydrogen bonding such that they are unavailable for anion binding and transport. Triggering using light (ONB) and reduction (azo) reverses the hydrogen bonding arrangement and generates a highly preorganized anion binding cavity for efficient transmembrane transport.

## 2 Results and Discussion

### 2.1 Synthesis and Anion Binding Studies of Active Transporters

**Scheme 1:**
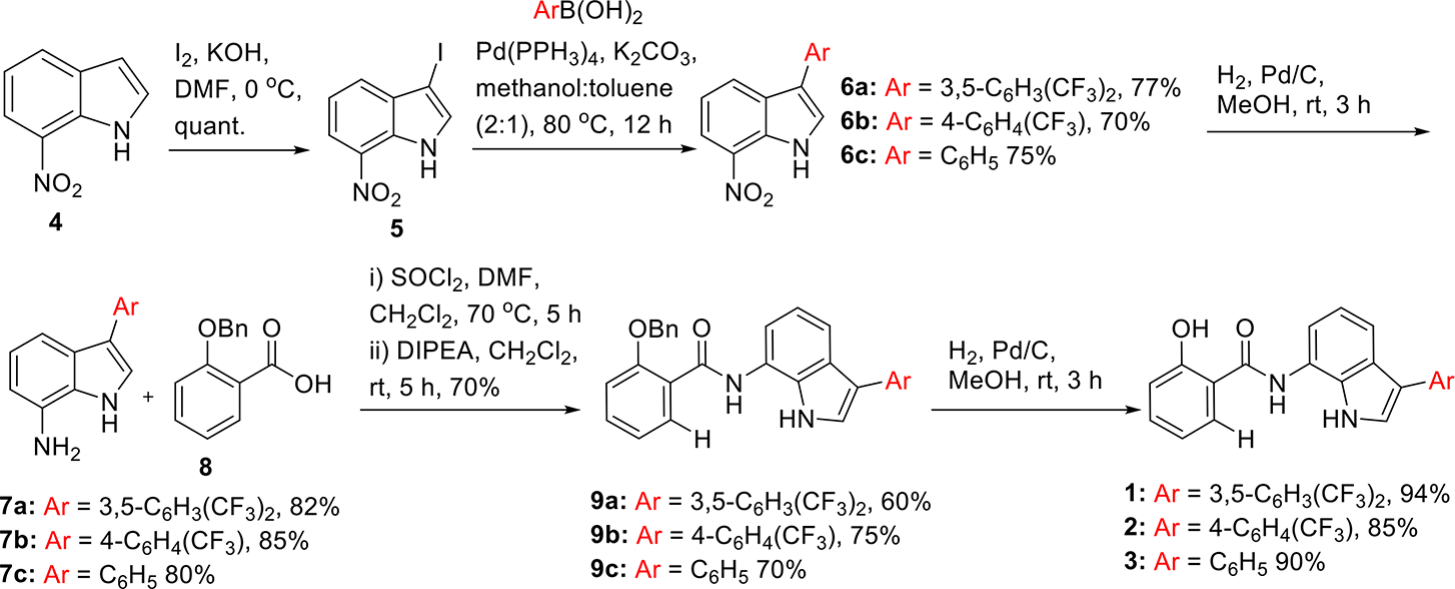
Chemical Synthesis of Active Transporters **1–3**

We designed and prepared a small library of three different transporters, **1–3** containing variable aromatic moieties. The nature of the aromatic groups was varied to tune the lipophilicity and binding affinity and optimize the permeability and transport affinity of these anionophores^6,44^ (Figure 1B). The synthesis of the active transporters **1**–**3** was achieved by iodination of **4** to form iodo-indole derivative **5**. Suzuki coupling of **5** with different aromatic boronic acids led to the formation of aromatic indole derivatives **6a–6c**, which upon subsequent hydrogenation gave amino derivatives **7a–7c**. Coupling **7a–7c** with benzylated salicylic acid **8** gave benzylated indole amide compounds **9a–9c**, which upon hydrogenation in the presence of Pd/C furnished active transporters **1–3** in excellent yield (Scheme 1).

**Figure 2:**
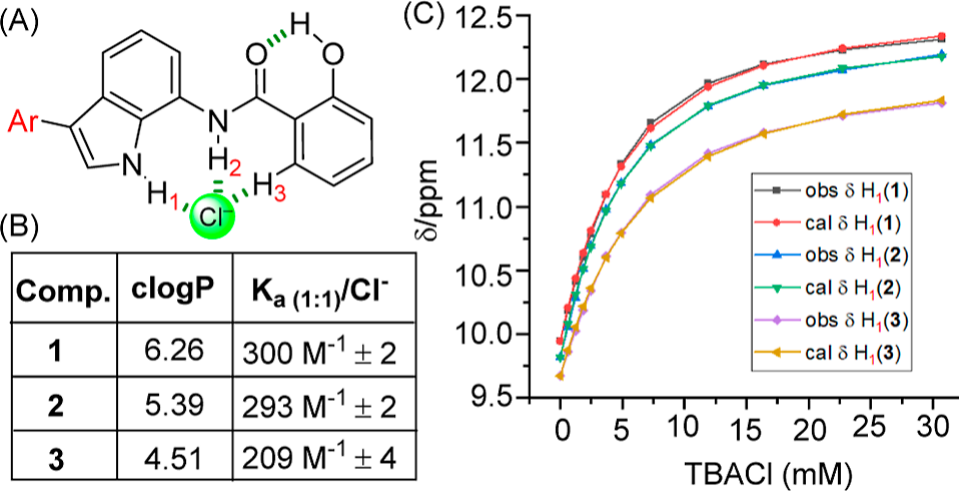
(A) General chemical structure representing transporters **1–3**. (B) Table showing clogP values (calculated using MarvinSketch^46^) and chloride association constant (*K*_a_) values of transporters **1–3**. (C) Plot of chemical shift (d) of H_1_ proton vs concentration of TBACl added, fitted to a 1:1 binding model, for the active transporters **1–3**.

The chloride binding capabilities of these derivatives were then examined by using ^1^H NMR titrations in acetonitrile-*d*_3_. Addition of tetrabutylammonium chloride (TBACl) to compounds **1–3** led to changes in H_1_, H_2_, and H_3_ protons, revealing their role in chloride binding through indole–N–H_1_···Cl^–^, amide–N–H_2_···Cl^–^, and ArC–H_3_···Cl^–^ hydrogen bonding interactions (Figures 2C, S35, S37, 39, and S41). The data could be fitted to a 1:1 host–guest binding model using BindFit,^45^ to determine association constant values (*K*_a(1:1)_/Cl^–^) of 300 M^–1^ ± 2% for **1**, 293 M^–1^ ± 2% for **2**, and 209 M^–1^ ± 4% for **3**, respectively (Figures 2B, S36, S38, S40, and S42).

### 2.2 Ion Transport Studies

**Figure 3:**
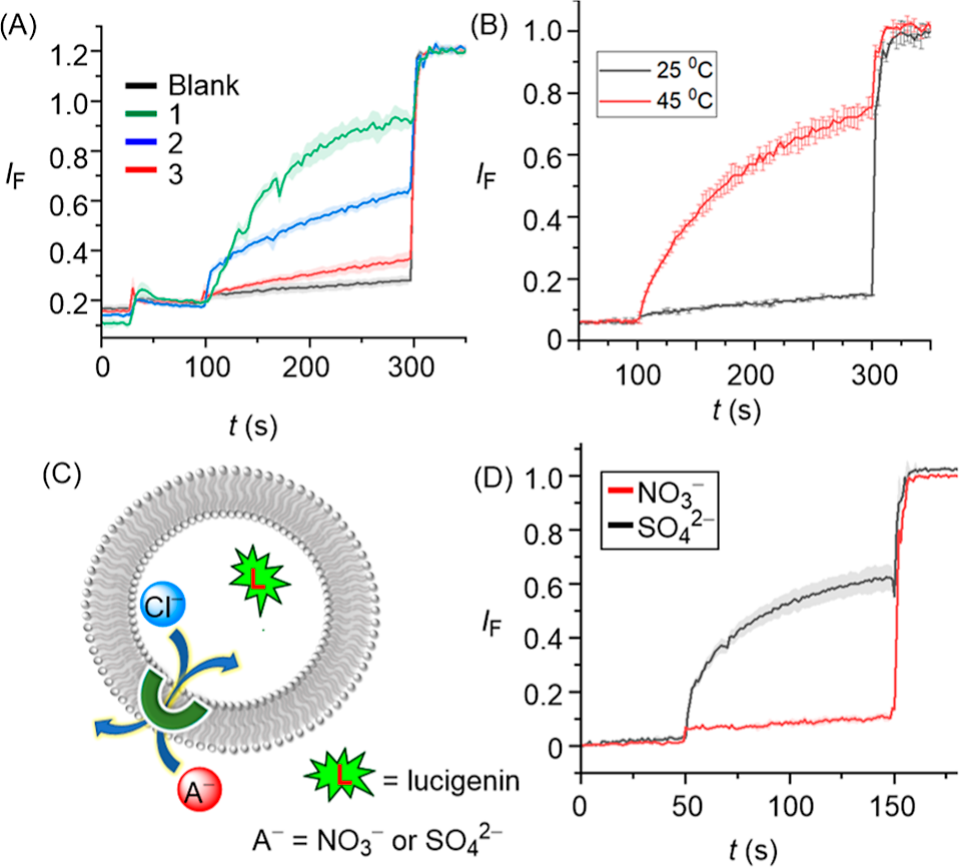
(A) Activity comparison of **1–3** (3.5 mol %) across POPC–LUVs⊃HPTS. (B) Ion transport activity of **1** (2.5 mol %) across DPPC-based vesicles at 25 and 45 °C, respectively. (C) Schematic representation of lucigenin-based chloride efflux using either extravesicular SO_4_^2–^ or NO_3_^–^ ions. (D) Ion transport activity of 1 (20 mol %) in the presence of external SO_4_^2–^ and NO_3_^–^ ions.

With evidence of appreciable chloride binding by **1–3** in hand, ion transport experiments were performed in large unilamellar vesicles (LUVs). LUVs were prepared using 1-palmitoyl-2-oleoyl-*sn*-glycero-3-phosphocholine lipid (POPC LUVs) encapsulating 8-hydroxypyrene-1,3,6-trisulfonate (HPTS, a ratiometric pH-sensitive dye) and buffered to pH 7.0 in NaCl solution. A pH gradient was created across the liposomal membrane by applying a NaOH pulse (0.5 M, 30 μL) in the extravesicular buffer. The ability of the anionophores to dissipate the pH gradient by transmembrane OH^–^/Cl^–^ antiport (or the functionally equivalent H^+^/Cl^–^ symport) was determined by recording the change in the HPTS emission, *I*_rel_ (λ_em_ = 510 nm), with time following excitation at λ_ex_ = 405/460 nm. At the end of each experiment, excess detergent (Triton X-100) was added to lyse the vesicles for calibration of the emission intensity. Significant ion transport was observed for all transporters, with an ion transport activity sequence of **1** > **2** > **3**. The activity of the three transporters at 3.5 mol % loading (with respect to lipid) is shown in Figure 3A, and the corresponding dose–response curves showing activity as a function of concentration are given in the ESI (Figures S45, S47, and S49). Hill analysis of this data furnished the EC_50_ values of 1.39 μM ± 0.09 (4.48 mol %) for **1** and 3.52 μM ± 0.23 (11.35 mol %) for **2**, respectively (Figures S46 and S48). Hill coefficients (*n*) were found to be ∼2 in both cases, indicative of the involvement of a 2:1 receptor–anion complex in the transmembrane anion transport process. Hill analysis could not be performed for transporter **3** due to precipitation issues at higher concentration (Figure S49). The above activity sequence of **1** > **2** > **3** is most likely determined by a combination of both the varying lipophilicities and chloride binding affinities of the transporters, with the best-performing transporter **1** featuring the highest chloride binding affinity and optimum logP value.

Mechanistically, in order to observe dissipation of the pH gradient in the HPTS assay, ion transport may occur in principle through (a) H^+^/X^–^ symport, (b) OH^–^/X^–^ antiport, (c) H^+^/M^+^ antiport, or (d) OH^–^/M^+^ symport modes. Variation of cations using different MCl salts in the external buffer (M = Li, Na, K, Rb, and Cs) did not affect the ion transport and hence ruled out the role of cations in the ion transport (Figure S51). The role of anions in the transport process was initially confirmed by varying the NaX salts in the external buffer (X = Cl, Br, I, ClO_4_, and NO_3_), which led to a significant change in the ion transport activity (Figure S50). Combined, these studies ruled out H^+^/M^+^ antiport or OH^–^/M^+^ symport ion transport modes and indicate that the ion transport occurs via either H^+^/X^–^ symport or OH^–^/X^–^ antiport mode, although the former is osmotically disfavored.

The transport process was further studied using a lucigenin-based fluorescence assay, in which lucigenin (a chloride-sensitive fluorophore) was encapsulated within LUVs containing NaCl (200 mM) buffered at a pH of 6.5. The effect of ion transport activity was monitored by adding either NaNO_3_ (200 mM) or Na_2_SO_4_ (200 mM) in the external buffer. The transport activity was determined by recording the change in the lucigenin emission, *I*_f_ (λ_em_ = 535 nm), with time following excitation at λ_ex_ = 460 nm. Significant transport activity was observed in the presence of NaNO_3_, which was absent in the presence of Na_2_SO_4_ (Figure 3D). The above results suggest Cl^–^/NO_3_^–^ antiport mode of ion transport in this assay, rather than H^+^/Cl^–^ symport, because in the latter, no change in transport rates would be expected by changing the anion, while for antiport, sulfate is highly hydrophilic and poorly transported compared to nitrate. Conducting analogous experiments in dipalmitoylphosphatidylcholine (DPPC) LUVs provided evidence for a mobile carrier mechanism. Inactivity at 25 °C, and restoration of activity at 45 °C, which is above the gel–liquid phase transition temperature for DPPC (*T*_m_ = 41 °C), is indicative of a mobile carrier process, rather than transport mediated by self-assembly into an ion channel, the activity of which would be typically expected to be independent of the lipid phase (Figure 3B) .

### 2.3 Synthesis and Anion Binding Studies of Caged Protransporters

**Scheme 2:**
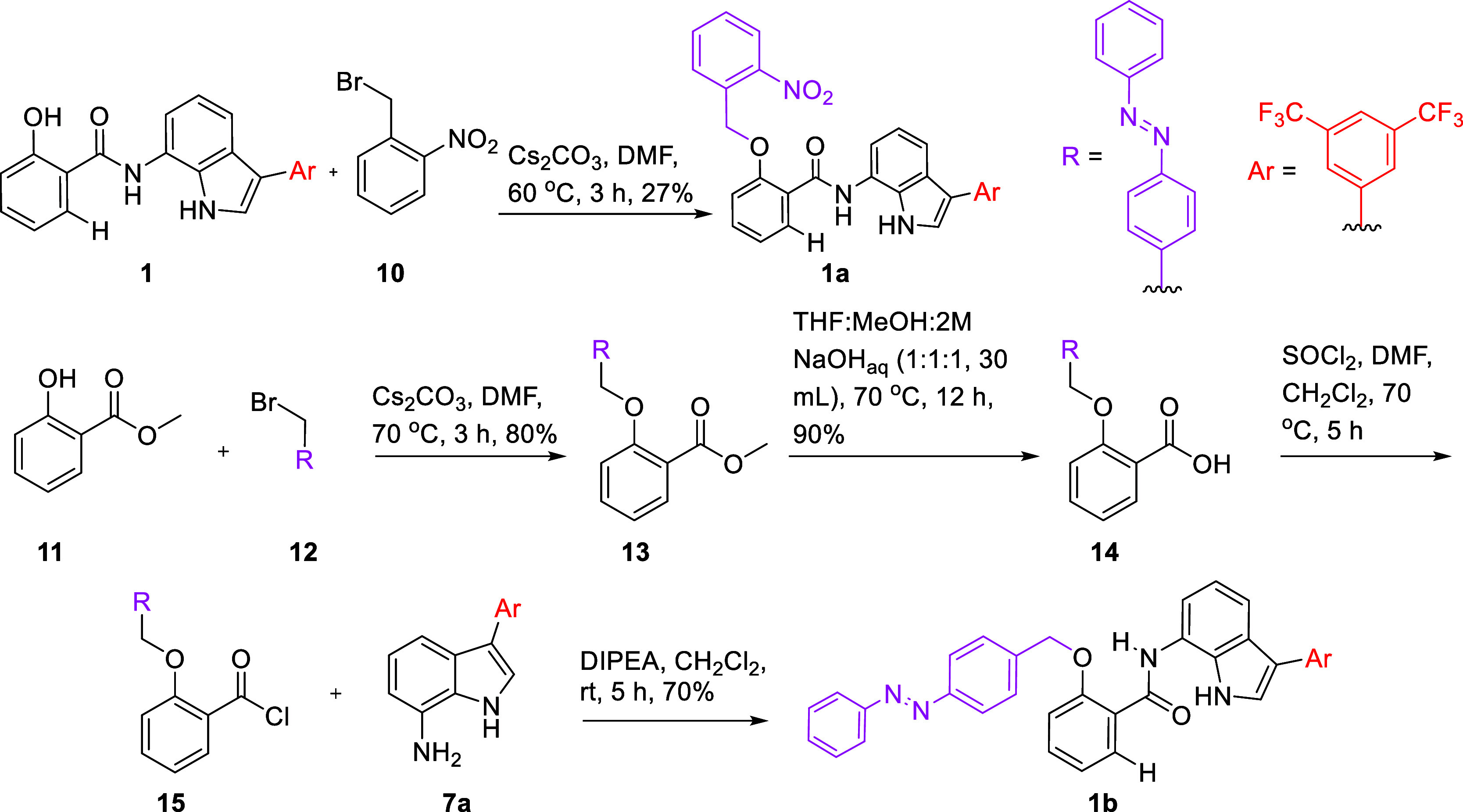
Chemical Synthesis of Inactive Protransporters **1a** and **1b**

Having demonstrated that compound **1** displayed the maximum ion transport activity, we selected this anionophore as the core scaffold for caging with *o*-nitrobenzyl and azobenzene stimulus-responsive groups. The synthesis of corresponding ONB-caged protransporter **1a** was achieved by treating active transporter **1** with 1-(bromomethyl)-2-nitrobenzene **10** in the presence cesium carbonate. To synthesize azobenzene-caged protransporter **1b**, 2-hydroxybenzoate **11** was treated with (*E*)-1-(4-(bromomethyl)phenyl)-2-phenyldiazene **12** to form azo derivative **13**, which upon hydrolysis gave carboxylic derivative **14**. Compound **14** was converted into acyl derivative **15** using thionyl chloride under reflux conditions. Finally, acyl derivative **15** was coupled with indole-amine compound **7a** to furnish azo-caged protransporter **1b** in good yield (Scheme 2). Following the synthesis of protransporters **1a** and **1b**, chloride anion binding NMR titration studies were performed in acetonitrile-*d*_3_ by addition of aliquots of TBACl. Both **1a** and **1b** displayed weak anion binding compared to active transporter **1** with association constant values of 112 M^–1^ ± 1% for **1a** and 63 M^–1^ ± 23% for **1b**, respectively (Figures S41–S44). The weak anion binding is the result of amide–N–H locking through six-membered intramolecular hydrogen bonding with the oxygen atom of the cage groups. In this state, indole NH is still available to bind anions, which presumably accounts for the weak anion affinity observed.

### 2.4 Stimulus-Responsive Activation

Stimulus-responsive activation studies on **1a** and **1b** were initially performed in solution-phase experiments. For **1a**, a solution of the protransporter in DMSO-*d*_6_ was subjected to photoirradiation using a 405 nm LED and analyzed by ^1^H NMR experiments (Figure S54). The changes in the chemical shifts of the protons are indicative of the photodecaging of protransporter **1a** to form active transporter **1**. The stimulus-responsive activation of **1b** on the other hand was performed by UV–vis absorption and HPLC analysis by addition of zinc metal in the presence of ammonium formate (see ESI for experimental details). The decrease in the absorption band at ∼350 nm upon reduction was indicative of the reduction of azo functionality; the change in the color of **1b** from a yellow to colorless solution further confirmed the azo reduction and could be observed by the naked eye (Figure S55). HPLC analysis provided direct proof of the reduction of **1b** to generate **1** (Figure S56).

**Figure 4:**
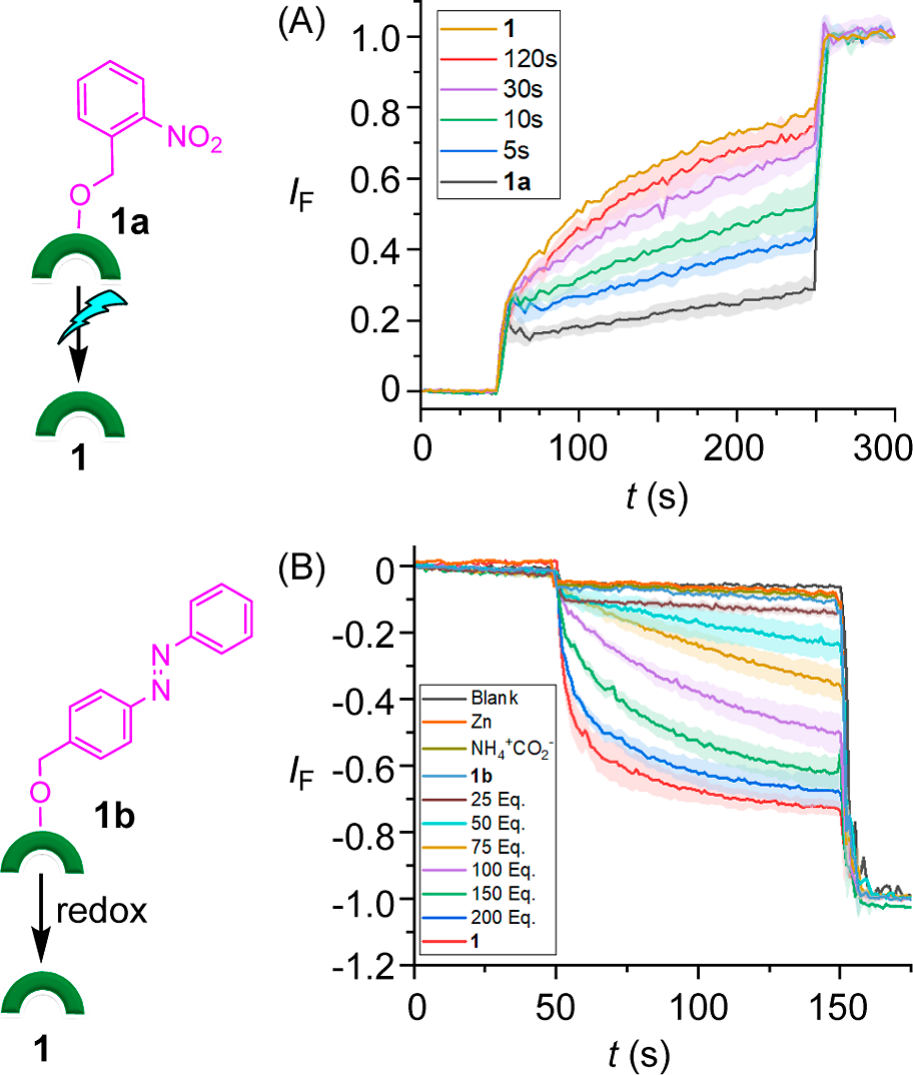
(A) Transport activity of **1a** upon photoirradiation at 405 nm of light using an LED (1 W) across POPC–LUVs⊃HPTS. (B) Transport activity of **1b** upon treating with zinc (10 equiv) and ammonium formate (25–200 equiv) across POPC–LUVs⊃lucigenin.

After the stimulus-responsive activation in the solution phase was successfully achieved, the triggered off–on activation of ion transport was subsequently evaluated. In the case of ONB-caged protransporter **1a**, this was examined using HPTS-containing LUVs. Addition of **1a** at 4.0 mol % loading did not lead to observable transport, thus constituting an excellent off state. In contrast, **1** at this loading led to rapid transport of the anion across the membrane. To explore in situ activation of **1a** to generate **1**, LUVs containing **1a** (4.0 mol %) were photoirradiated at 405 nm using an LED (1 W) for different time intervals and ion transport was monitored after each photoirradiation process. A significant enhancement in the ion transport activity was observed upon photoirradiation, indicative of formation of active transporter **1** (Figure 4A).

For **1b**, stimulus-responsive activation was achieved using lucigenin-based LUVs that report on chloride/nitrate exchange because ammonium formate itself interferes with HPTS fluorescence. Before the actual activation of **1b**, dose-dependent transport activity of **1** was performed using LUVs containing 1 mM lucigenin (in 200 mM sodium nitrate buffered to pH 6.5 with 10 mM phosphate buffer) to determine the optimum concentration required for the activation process. Ion transport was monitored by creating a 33.3 mM Cl^–^ gradient across the membrane by adding a NaCl pulse (50 μL, 0.5 M) to the extravesicular buffer, and the rate of fluorescence quenching for lucigenin was recorded upon the addition of transporters **1**. The concentration-dependent studies are shown in Figure S57. From these experiments, we selected a 30 mol % transporter loading at which caged derivative **1b** was inactive, and the corresponding active transporter **1** displayed significant transport activity. To generate **1** from **1b**, a sample of **1b** in 2:1 MeOH/THF was reduced in the presence of zinc and ammonium formate, before addition of aliquots of this solution to the fluorescence cuvette containing 200 mM NaNO_3_ (2910 μL) and lucigenin-based vesicles (20 μL, 54.8 μM). Significant enhancement in the ion transport was observed, which increased as a function of formate concentration, indicative of the reduction of the azobenzene subunit and the generation of transporter **1** (Figure 4B). To the best of our knowledge, this is the first example of utilizing this azo-reduction methodology—well-established for hypoxia-triggered prodrugs—to activate ionophores.

## 3 Conclusions

In conclusion, we have developed indole-derived multistimulus-responsive anion transporters that are activated by both light and redox external stimuli. Azobenzene and ONB cages rendered the indole-amide-based transporters inactive by locking the amide proton through intramolecular hydrogen bonding, while decaging led to the reversal of hydrogen bonding, which reveals the amide NH protons for anion binding and transport. This work demonstrates that azobenzene-based hypoxia-responsive cages are amenable for triggered off–on activation of anion transport, opening up the possibility of activation in tumors using hypoxia-induced reductase enzymes. We anticipate that this work could provide a scaffold with which to develop ionophores with optimum solubility and efficient reactivity profiles that are amenable to such in situ, in-tumor activation studies in future.

## 4 Experimental Section

### 4.1 Materials and Methods

All reagents and solvents were purchased from commercial sources and used without further purification. Lipids were purchased from Avanti polar lipids and used without further purification. Column chromatography was carried out on Merck silica gel 60 under a positive pressure of nitrogen, where mixtures of solvents were used and ratios are reported by volume. NMR spectra were recorded on Bruker AVIII 400, Bruker AVII 500 (with a cryoprobe), and Bruker AVIII 500 spectrometers. Chemical shifts are reported as δ values in ppm, referring to the residual deuterated solvent signals. Mass spectra were carried out on Waters Micromass LCT and Bruker microTOF spectrometers. UV–vis spectra were recorded on a Jasco V-770 UV–visible/NIR spectrophotometer equipped with a Peltier temperature controller and stirrer using quartz cuvettes of 1 cm path length. Experiments were conducted at 25 °C unless otherwise stated. HPLC analyses were carried out using a Thermo Scientific Vanquish Core HPLC system and a C-18 reverse phase column (Ascentis, 5 μm, 15 cm × 4.6 mm).

### 4.2 Anion Binding Studies

TBACl and the receptor were dried under high vacuum before use. The titrations were performed by the addition of aliquots from the solution of TBACl (0.25 M in CD_3_CN) to the solution of receptors either of **1**, **2**, **3**, **1a**, or **1b** (2.5 mM), respectively. All NMR data were processed using MestReNova 6.0, and the collected data were analyzed using BindFit v0.5.

### 4.3 Vesicle Preparation and Anion Transport Assays

A thin film of lipid (POPC or DPPC) was formed by evaporating a chloroform solution under a stream of nitrogen gas and then under high vacuum for 6 h. The lipid film was hydrated by vortexing with the prepared buffer (100 mM NaCl, 10 mM HEPES, 1 mM 8-hydroxypyrene-1,3,6-trisulfonic acid trisodium salt (HPTS), pH 7.0) and subjected to 5 freeze–thaw cycles using liquid nitrogen and a water bath (40 °C), followed by extrusion 19 times through a polycarbonate membrane (pore size 200 nm) at rt using an Avestin “LiposoFast” extruder. Extrusion was performed at 50 °C in the case of DPPC lipids. Extravesicular components were removed by size exclusion chromatography on a Sephadex G-25 column with 100 mM NaCl and 10 mM HEPES, pH 7.0. Final conditions: LUVs (2.5 mM lipid); inside: 100 mM NaCl, 10 mM HEPES, 1 mM HPTS, pH 7.0; outside: 100 mM NaCl, 10 mM HEPES, pH 7.0. For LUVs containing lucigenin, the same procedure was followed, except that the lipids were hydrated with aqueous NaNO_3_ (200 mM, 1.0 mM lucigenin) in 10 mM phosphate buffer at pH 6.5.

Anion transport experiments were conducted using an Agilent Cary Eclipse fluorescence spectrophotometer, equipped with a Peltier temperature controller and stirrer. In a typical experiment, the LUVs containing HPTS (40 μL, final lipid concentration 31.3 μM) were added to buffer (2910 μL of 100 mM NaCl and 10 mM HEPES, pH 7.0) at 25 °C under gentle stirring. A pulse of NaOH (30 μL, 0.5 M) was added at 20 s to initiate the experiment. At 100 s, the test transporter was added, followed by detergent (40 μL of Triton X-100 in 7:1 (v/v) H_2_O–DMSO) at 300 s to calibrate the assay. The fluorescence emission was monitored at λ_em_ = 510 nm (λ_ex_ = 460/405 nm). For assays with lucigenin vesicles, the fluorescence intensity of lucigenin was monitored at λ_em_ = 535 nm (λ_ex_ = 455 nm). Detailed experimental procedures for these transport assays are available in the ESI.

### 4.4 Experimental Procedures for Synthesis

#### 4.4.1 3-Iodo-7-nitro-1*H*-indole, C_8_H_5_N_2_O_2_ (**5**)

In a 25 mL round-bottom flask, compound **4** (1g, 6.2 mM, 1 equiv) was dissolved in DMF (10 mL). The solution was kept at 0 °C and KOH (2.08 g, 37 mmol, 10 equiv) was added, and after 10 min, iodine (1.88, 7.4 mmol, 1.2 equiv) dissolved in DMF was added dropwise. The reaction was allowed to stir at rt for 2 h. After the completion of the reaction, the mixture was poured into a solution of aqueous ammonium chloride and sodium thiosulfate. The yellow precipitate was collected by vacuum filtration and dried under vacuum to furnish compound **5** in quantitative yield (1.75 g). ^1^H NMR (400 MHz, CDCl_3_): δ 10.08 (s, 1H), 8.24 (d, *J* = 8.1 Hz, 1H), 7.83 (d, *J* = 7.8 Hz, 1H), 7.50 (d, *J* = 2.4 Hz, 1H), 7.31 (t, *J* = 7.9 Hz, 1H). ^13^C NMR (151 MHz, CDCl_3_): δ 133.3, 133.0, 130.9, 129.3, 129.2, 120.2, 119.9, 58.9; HRMS- ESI for [M + H]^+^, C_8_H_5_N_2_O_2_ calculated 287.9156; found, 287.9134.

#### 4.4.2 General Procedure for the Synthesis of **6a–6c**

3-Iodo-7-nitroindole **5** (0.5 g, 1.7 mmol, 1 equiv) and the corresponding aryl boronic acid (2.6 mmol, 1.5 equiv) were dissolved in a mixture of degassed MeOH (20 mL) and toluene (10 mL) under N_2_. A solution of Pd(PPh_3_)_4_ (0.032 g, 0.086 mmol, 0.05 equiv) in Na_2_CO_3_ (2 M, 10 mL) was added and the solution was refluxed for 12 h at 70 °C. The catalyst was removed by Celite filtration washing with EtOAc. The product was extracted with EtOAc (30 mL) and washed with brine (30 mL). The organic phase was separated and dried with MgSO_4_. The product was purified by silica column chromatography to furnish compound **6a–6c** in excellent yields.

#### 4.4.3 3-(3,5-Bis(trifluoromethyl)phenyl)-7-nitro-1*H*-indole, C_16_H_8_F_6_N_2_O_2_ (**6a**)

This compound was synthesized by reacting 3-iodo-7-nitro-1*H*-indole **5** (0.5 g, 1.7 mmol, 1 equiv) with (3,5-bis(trifluoromethyl)phenyl)boronic acid (0.67 g, 2.6 mmol, 1.5 equiv). Yield: 77%, 355 mg. ^1^H NMR (600 MHz, CDCl_3_): δ 10.21 (s, 1H), 8.28 (d, *J* = 8.0 Hz, 1H), 8.17 (d, *J* = 7.8 Hz, 1H), 8.05 (s, 2H), 7.86 (s, 1H), 7.67 (d, *J* = 2.5 Hz, 1H), 7.38 (t, *J* = 8.0 Hz, 1H); ^13^C NMR (126 MHz, CDCl_3_): δ 136.3, 133.5, 132.5, 130.1, 129.0, 127.7, 127.2, 125.2, 123.4, 120.7, 120.5, 120.4, 117.3. HRMS- ESI for [M + H]+, C_16_H_8_F_6_N_2_O_2_ calculated 397.0382; found, 397.0362.

#### 4.4.4 7-Nitro-3-(4-(trifluoromethyl)phenyl)-1*H*-indole, C_15_H_9_F_3_N_2_O_2_ (**6b**)

This compound was synthesized by reacting 3-iodo-7-nitro-1*H*-indole **5** (0.5 g, 1.7 mmol, 1 equiv) with (3,5-(4-(trifluoromethyl)phenyl)boronic acid (0.5 g, 2.6 mmol, 1.5 equiv). Yield: 70%, 370 mg. ^1^H NMR (600 MHz, CDCl_3_): δ 10.16 (s, 1H), 8.26 (dd, *J* = 16.9, 8.5 Hz, 2H), 7.76 (s, 4H), 7.63 (s, 1H), 7.35 (t, *J* = 7.9 Hz, 1H); ^13^C NMR (151 MHz, CDCl_3_): δ 137.6, 133.3, 130.0, 129.3, 129.2–128.6 (q) 127.9, 127.6, 126.9–121.5 (q), 126.02–125.9 (q), 124.6, 120.1, 120.0, 118.5; HRMS- ESI for [M + H]^+^, C_15_H_9_F_3_N_2_O_2_ calculated 307.0689; found, 307.0682.

#### 4.4.5 7-Nitro-3-phenyl-1*H*-indole, C_14_H_10_N_2_O_2_ (**6c**)

This compound was synthesized by reacting 3-iodo-7-nitro-1*H*-indole **5** (0.5 g, 1.7 mmol, 1 equiv) with (3,5-(4-(trifluoromethyl)phenyl)boronic acid (0.32 g, 2.6 mmol, 1.5 equiv). Yield: 75%, 309 mg. ^1^H NMR (400 MHz, CDCl_3_): δ 10.04 (s, 1H), 8.27–8.20 (m, 2H), 7.66–7.60 (m, 2H), 7.54 (d, *J* = 2.4 Hz, 1H), 7.49 (t, *J* = 7.6 Hz, 2H), 7.37 (t, *J* = 7.4 Hz, 1H), 7.29 (d, *J* = 7.9 Hz, 1H); ^13^C NMR (126 MHz, CDCl_3_): δ 134.0, 133.3, 130.1, 129.9, 129.2, 128.2, 128.1, 127.1, 124.0, 120.0, 119.9, 119.8; HRMS-ESI for [M + H]^+^, C_14_H_10_N_2_O_2_ calculated 239.0815; found, 239.0814.

### 4.5 General Procedure for the Synthesis of **7a–7c**

In a 25 mL round-bottom flask, compound **6a–6c** (300 mg) was dissolved in ethanol (30 mL) to which 10% Pd/C (20 mg) was added. The reaction mixture was subjected to stirring for a duration of 3 h under a hydrogen gas atmosphere. Following the reaction, the Pd/C was filtered out, and the resulting filtrate was collected and dried under vacuum to yield a crude product that was washed with pentane and diethyl ether to furnish compounds **7a–7c** in 80–85% yield.

#### 4.5.1 3-(3,5-Bis(trifluoromethyl)phenyl)-1*H*-indol-7-amine, C_16_H_10_F_6_N_2_ (**7a**)

The compound **7a** was obtained as a light-gray solid (225 mg, 82%). ^1^H NMR (600 MHz, DMSO-*d*_6_): δ 11.33 (s, 1H), 8.24 (s, 2H), 8.04 (d, *J* = 2.9 Hz, 1H), 7.84 (s, 1H), 7.09 (d, *J* = 7.9 Hz, 1H), 6.92 (t, *J* = 7.8 Hz, 1H), 6.45 (d, *J* = 6.5 Hz, 1H), 5.19 (s, 2H); ^13^C NMR (126 MHz, DMSO-*d*_6_): δ 139.2, 134.6, 130.8, 126.4, 125.6, 125.0, 124.7, 123.6, 122.0, 117.6, 112.9, 106.3, 105.6; HRMS-ESI for [M + H]^+^, C_16_H_10_F_6_N_2_ calculated 345.0821; found, 345.0840.

#### 4.5.2 3-(4-(Trifluoromethyl)phenyl)-1*H*-indol-7-amine, C_15_H_11_F_3_N_2_ (**7b**)

The compound **7b** was obtained as a light-gray solid (224 mg, 85%). ^1^H NMR (600 MHz, DMSO-*d*_6_): δ 11.15 (s, 1H), 7.89 (d, *J* = 8.2 Hz, 2H), 7.81 (d, *J* = 2.8 Hz, 1H), 7.73 (d, *J* = 8.2 Hz, 2H), 7.19 (d, *J* = 8.2 Hz, 1H), 6.89 (t, *J* = 7.7 Hz, 1H), 6.44 (dt, *J* = 7.4, 1.0 Hz, 1H), 5.24 (s, 2H); ^13^C NMR (151 MHz, DMSO): δ 141.2, 134.6, 127.8–122.1 (q), 127.0, 126.7, 126.0–125.9 (q), 125.8, 125.7–125.1 (q) 124.1, 121.8, 115.0, 107.8, 106.0; HRMS-ESI for [M + H]^+^, C_15_H_11_F_3_N_2_ calculated 277.0947; found, 277.0978.

#### 4.5.3 3-Phenyl-1*H*-indol-7-amine, C_14_H_12_N_2_ (**7c**)

The compound **7c** was obtained as a dark-gray solid (209 mg, 80%). ^1^H NMR (400 MHz, DMSO): δ 10.90 (s, 1H), 7.65 (d, *J* = 7.9 Hz, 2H), 7.60 (d, *J* = 2.7 Hz, 1H), 7.40 (t, *J* = 7.8 Hz, 2H), 7.19 (t, *J* = 7.4 Hz, 1H), 7.11 (d, *J* = 7.9 Hz, 1H), 6.82 (t, *J* = 7.7 Hz, 1H), 6.38 (d, *J* = 7.3 Hz, 1H), 5.09 (s, 2H); ^13^C NMR (126 MHz, CDCl_3_): δ 136.4, 134.2, 128.6, 126.4, 126.3, 125.6, 124.9, 122.0, 120.8, 116.1, 107.4, 105.1; HRMS-ESI for [M + H]+, C_14_H_12_N_2_ calcd 209.1073; found, 209.1077.

### 4.6 General Procedure for the Synthesis of **9a–9c**

In a 25 mL round-bottom flask, 2-(benzoyloxy) benzoic acid **8**(47) (200 mg, 0.87 mmol, 1 equiv) was dissolved in dry dichloromethane (30 mL). To this mixture, thionyl chloride (1.9 g, 20 equiv) and few drops of dry DMF were added, and the reaction was refluxed at 70 °C for 5 h. The solvent was then evaporated using a rotary evaporator to give the crude mixture which was further concentrated twice with dry dichloromethane to remove excess of thionyl chloride. The white precipitate obtained was redissolved in dry dichloromethane and added slowly to either **7a**, **7b**, or **7c** (1 equiv) solutions dissolved in dry dichloromethane. DIPEA (0.14 mL, 0.87 mmol, 1 equiv) was added and the reaction mixture was allowed to stir at rt for further 3 h. After the completion of the reaction, the solvent was evaporated using a rotary evaporator, and the crude mixture was extracted with ethyl acetate (3 × 30 mL). The organic layers were dried with anhydrous magnesium sulfate, filtered, and concentrated under reduced pressure to give the crude product. The crude residue was then purified by column chromatography over silica gel to furnish **9a–9c** in 60–75% yields.

#### 4.6.1 2-(Benzyloxy)-*N*-(3-(3,5-bis(trifluoromethyl)phenyl)-1*H*-indol-7-yl)benzamide, C_30_H_20_F_6_N_2_O_2_ (**9a**)

This compound was synthesized by reacting 2-(benzoyloxy)benzoic acid **8** (200 mg, 0.82 mmol, 1 equiv) with **7a** (301 mg, 0.87 mmol, 1 equiv) and the crude product was purified by column chromatography over silica gel (eluent: 30% ethyl acetate in hexane) to obtain **9a** as a light-green solid. Yield: 60%, 291 mg. ^1^H NMR (600 MHz, CDCl_3_): δ 10.83 (s, 1H), 10.43 (s, 1H), 8.40 (dd, *J* = 7.8, 1.8 Hz, 1H), 8.05 (s, 2H), 7.74 (s, 1H), 7.63–7.55 (m, 4H), 7.51 (dd, *J* = 5.0, 2.0 Hz, 3H), 7.47 (d, *J* = 2.6 Hz, 1H), 7.25–7.19 (m, 2H), 6.94 (t, *J* = 7.8 Hz, 1H), 6.10 (d, *J* = 6.8 Hz, 1H), 5.29 (s, 2H). ^13^C NMR (126 MHz, CDCl_3_): δ 163.0, 157.1, 138.2, 135.0, 134.0, 132.8, 132.0, 129.5, 129.4, 128.9, 128.9, 127.8, 127.1, 123.9, 123.7, 123.6, 122.2, 121.0, 120.7, 119.1, 115.8, 115.3, 114.0, 112.8, 72.2; HRMS-ESI for [M + H]^+^ C_30_H_20_F_6_N_2_O_2_ calculated 555.1502; found, 555.1513.

#### 4.6.2 2-(Benzyloxy)-*N*-(3-(4-(trifluoromethyl)phenyl)-1*H*-indol-7-yl)benzamide, C_29_H_21_F_3_N_2_O_2_ (**9b**)

This compound was synthesized by reacting 2-(benzoyloxy)benzoic acid **8** (200 mg, 0.82 mmol, 1 equiv) with **7b** (194 mg, 0.87 mmol, 1 equiv) and the crude product was purified by column chromatography over silica gel (eluent: 30% ethyl acetate in hexane) to obtain **9b** as a green solid. Yield: 75%, 319 mg. ^1^H NMR (600 MHz, CDCl_3_): δ 10.65 (s, 1H), 10.42 (s, 1H), 8.42 (dd, *J* = 7.8, 1.8 Hz, 1H), 7.77 (d, *J* = 8.7 Hz, 2H), 7.70 (dd, *J* = 8.0, 4.8 Hz, 3H), 7.61–7.58 (m, 3H), 7.53 (dd, *J* = 4.6, 2.1 Hz, 3H), 7.45 (d, *J* = 2.5 Hz, 1H), 7.26–7.21 (m, 2H), 6.94 (t, *J* = 7.8 Hz, 1H), 6.14 (d, *J* = 6.7 Hz, 1H), 5.31 (s, 2H); ^13^C NMR (151 MHz, CDCl_3_): δ 162.8, 156.9, 139.6, 133.7, 132.7, 129.3, 129.3, 128.8, 128.8, 128.0, 127.8–127.2 (q), 127.3, 127.2–121.8 (q), 125.7–121.8 (q), 123.5, 123.2, 122.0, 121.1, 120.1, 116.6, 116.3, 113.7, 112.7, 72.0; HRMS-ESI for [M + H]^+^ C_29_H_21_F_3_N_2_O_2_ calculated 487.1628; found, 487.1640.

#### 4.6.3 2-(Benzyloxy)-*N*-(3-phenyl-1*H*-indol-7-yl)benzamide, C_28_H_22_N_2_O_2_ (**9c**)

This compound was synthesized by reacting 2-(benzoyloxy)benzoic acid **8** (200 mg, 0.82 mmol, 1 equiv) with **7c** (182 mg, 0.87 mmol, 1 equiv) and the crude product was purified by column chromatography over silica gel (eluent: 30% ethyl acetate in hexane) to obtain **9c** as a light-green solid. Yield: 70%, 256 mg. ^1^H NMR (600 MHz, CDCl_3_): δ 10.36 (d, *J* = 11.5 Hz, 2H), 8.40 (dd, *J* = 7.8, 1.9 Hz, 1H), 7.71 (d, *J* = 9.0 Hz, 1H), 7.65 (dd, *J* = 8.3, 1.3 Hz, 2H), 7.57 (td, *J* = 6.7, 2.2 Hz, 3H), 7.52–7.47 (m, 3H), 7.44 (t, *J* = 7.8 Hz, 2H), 7.36 (s, 1H), 7.28 (t, *J* = 7.4 Hz, 1H), 7.24–7.18 (m, 2H), 6.89 (t, *J* = 7.7 Hz, 1H), 6.17 (d, *J* = 8.4 Hz, 1H), 5.28 (s, 2H); ^13^C NMR (151 MHz, CDCl_3_): δ 162.8, 157.0, 135.9, 135.1, 133.7, 132.8, 129.4, 129.4, 128.9, 128.9, 128.8, 128.5, 127.7, 125.9, 123.4, 122.6, 122.1, 121.4, 119.8, 118.1, 116.8, 113.6, 112.8, 72.1; HRMS-ESI for [M + H]^+^ C_28_H_22_N_2_O_2_ calculated 419.1754; found, 419.1756.

### 4.7 General Procedure for the Synthesis of **1–3**

In a 25 mL round-bottom flask, **9a–9c** (100 mg) was dissolved in THF/methanol (2:1, 30 mL) to which 10% Pd/C (20 mg) was added. The reaction was subjected to stirring for a duration of 3 h under a hydrogen gas atmosphere. Following the reaction, the Pd/C was filtered out, and the resulting filtrate was collected and dried under vacuum to yield a crude product that was washed with pentane and diethyl ether to furnish compounds **1–3** in 85–94% yields.

#### 4.7.1 *N*-(3-(3,5-Bis(trifluoromethyl)phenyl)-1*H*-indol-7-yl)-2-hydroxybenzamide, C_23_H_14_F_6_N_2_O_2_ (**1**)

The compound **1** was obtained as a light-gray solid (63 mg, 94%). ^1^H NMR (600 MHz, DMSO-*d*_6_): δ 12.23 (s, 1H), 11.67 (s, 1H), 10.74 (s, 1H), 8.31 (s, 2H), 8.14–8.08 (m, 2H), 7.91 (s, 1H), 7.78 (d, *J* = 8.0 Hz, 1H), 7.51–7.44 (m, 1H), 7.32 (d, *J* = 7.4 Hz, 1H), 7.22 (t, *J* = 7.8 Hz, 1H), 6.98 (d, *J* = 8.2 Hz, 2H); ^13^C NMR (126 MHz, DMSO-*d*_6_): δ 168.0, 160.3, 138.5, 133.9, 131.8, 130.8, 128.8, 126.3, 126.1, 125.9, 123.5, 122.6, 120.6, 118.3, 118.2, 117.4, 116.3, 116.2, 113.0; HRMS-ESI for [M + H]^+^ C_23_H_14_F_6_N_2_O_2_ calculated 465.1032; found, 465.1027.

#### 4.7.2 2-Hydroxy-*N*-(3-(4-(trifluoromethyl)phenyl)-1H-indol-7-yl)benzamide, C_22_H_15_F_3_N_2_O_2_ (**2**)

The compound **2** was obtained as a white solid (68 mg, 85%). ^1^H NMR (500 MHz, CD_3_CN): δ 12.18 (s, 1H), 9.82 (s, 1H), 9.25 (s, 1H), 7.98 (d, *J* = 7.8 Hz, 1H), 7.93 (d, *J* = 6.4 Hz, 3H), 7.79 (d, *J* = 8.2 Hz, 2H), 7.68 (s, 1H), 7.55 (t, *J* = 7.9 Hz, 1H), 7.26 (d, *J* = 5.2 Hz, 2H), 7.06 (t, *J* = 8.5 Hz, 2H); ^13^C NMR (126 MHz, DMSO): δ 167.5, 164.5, 140.1, 133.2, 130.7, 129.1, 126.6, 126.3, 125.6, 125.3, 124.9, 124.6, 124.1, 120.3, 119.0, 117.0, 116.0, 115.5, 115.0, 114.5; HRMS-ESI for [M + H]^+^ C_22_H_15_F_3_N_2_O_2_ calculated 397.1158; found, 397.1177.

#### 4.7.3 2-Hydroxy-*N*-(3-phenyl-1*H*-indol-7-yl)benzamide, C_21_H_16_N_2_O_2_ (**3**)

The compound **3** was obtained as a white solid (70 mg, 90%). ^1^H NMR (400 MHz, CDCl_3_): δ 11.79 (s, 1H), 9.48 (s, 1H), 8.31 (s, 1H), 7.87 (d, *J* = 8.6 Hz, 1H), 7.69–7.61 (m, 3H), 7.54–7.42 (m, 4H), 7.31 (t, *J* = 7.4 Hz, 1H), 7.18 (t, *J* = 7.8 Hz, 1H), 7.09 (d, *J* = 8.4 Hz, 1H), 7.02–6.95 (m, 2H); ^13^C NMR (151 MHz, DMSO-*d*_6_): δ 168.3, 136.1, 134.1, 131.8, 129.3, 129.2, 127.1, 127.0, 125.8, 124.0, 123.0, 120.0, 118.2, 117.6, 117.2, 116.9, 116.6; HRMS-ESI for [M + H]^+^ C_21_H_16_N_2_O_2_ calculated 329.1285; found, 329.1274.

### 4.8 Synthesis of ONB-Protected Caged Compound **1a**

Compound **1** (40 mg, 0.086 mmol, 1 equiv) was dissolved in anhydrous DMF (5 mL) along with compound **10** (17.3 mg, 0.080 mmol, 0.93 equiv) and Cs_2_CO_3_ (28 mg, 0.086 mmol, 1 equiv). The reaction mixture was stirred at 60 °C for 3 h. The product was extracted with EtOAc (30 mL) and washed with brine (30 mL), the organic layers were collected, and the samples were dried with MgSO_4_. The product was isolated by silica flash chromatography using a 10% up to 25% EtOAc in hexane solvent system. The product was isolated as a green solid (14 mg, 27.2%). ^1^H NMR (400 MHz, CDCl_3_): δ 10.78 (s, 1H), 10.05 (s, 1H), 8.35 (d, *J* = 6.6 Hz, 1H), 8.23 (d, *J* = 8.1 Hz, 1H), 8.08 (s, 2H), 7.88–7.45 (m, 8H), 7.08 (dt, *J* = 15.0, 7.7 Hz, 2H), 6.54 (d, *J* = 7.3 Hz, 1H), 5.77 (s, 2H); ^13^C NMR (126 MHz, CDCl_3_): δ 163.2, 156.2, 147.7, 138.1, 134.5, 134.0, 132.9, 131.5, 129.8, 129.6, 129.1, 128.0, 127.2, 125.7, 123.9, 123.6, 122.8, 121.8, 120.8, 119.2, 116.3, 115.5, 114.2, 113.3, 68.6; HRMS-ESI for [M + H]^+^ C_30_H_19_F_6_N_3_O_3_ calculated 622.1172; found, 622.1184.

#### 4.8.1 Methyl (*E*)-2-((4-(phenyldiazenyl)benzyl)oxy)benzoate, C_21_H_18_N_2_O_3_ (**13**)

In a 25 mL round-bottom flask, methyl 2-hydroxybenzoate **11** (200 mg, 1.31 mmol, 1 equiv), (*E*)-1-(4-(bromomethyl)phenyl)-2-phenyldiazene **12** (361 mg, 1.31 mmol, 1 equiv), which itself was synthesized using the reported literature, and cesium carbonate (179 mg, 1.31 mmol, 1 equiv) were dissolved in DMF (10 mL). The reaction mixture was stirred at 70 °C for 3 h. The product was extracted with EtOAc (30 mL) and washed with brine (30 mL), collecting the organic layers and drying with MgSO_4_. The product was isolated by silica flash chromatography using a 20% EtOAc in hexane solvent system. The product was isolated as an orange solid (364 mg, 85%). ^1^H NMR (400 MHz, CDCl_3_): δ 7.90–7.82 (m, 4H), 7.77 (dd, *J* = 7.9, 1.8 Hz, 1H), 7.57 (d, *J* = 8.7 Hz, 2H), 7.47–7.34 (m, 4H), 6.96–6.91 (m, 2H), 5.18 (s, 2H), 3.85 (s, 3H); ^13^C NMR (151 MHz, CDCl_3_): δ 165.8, 157.0, 151.7, 151.3, 138.9, 132.6, 131.0, 130.1, 128.2, 126.5, 122.2, 121.9, 119.9, 119.9, 69.2, 51.2; HRMS-ESI for [M + H]^+^ C_21_H_18_N_2_O_3_ calculated 369.1210; found, 369.1220.

#### 4.8.2 (*E*)-2-((4-(Phenyldiazenyl)benzyl)oxy)benzoic acid, C_20_H_16_N_2_O_3_ (**14**)

A solution of **13** (350 mg, 1.01 mmol, 1 equiv) in a mixture of THF/MeOH/2 M aq. NaOH (1:1:1, 30 mL) was heated under reflux conditions using an oil bath for 12 h. After cooling to room temperature, the reaction mixture was poured into cold water (60 mL) and the pH of the solution was adjusted to 1.0 using concentrated HCl. The reaction mixture was then extracted with ethyl acetate (2 × 50 mL), washed with brine, and dried over magnesium sulfate. The solvent was evaporated under reduced pressure, and the crude mixture was purified by column chromatography over silica gel (eluent: 5% MeOH in CHCl_3_), to furnish the compound **13** as an orange solid (315 mg, 90%). ^1^H NMR (400 MHz, DMSO-*d*_6_): δ 12.71 (s, 1H), 7.92 (t, *J* = 8.4 Hz, 4H), 7.74 (d, *J* = 8.6 Hz, 2H), 7.69 (dd, *J* = 7.7, 1.8 Hz, 1H), 7.65–7.57 (m, 3H), 7.52 (t, *J* = 7.0 Hz, 1H), 7.23 (d, *J* = 8.1 Hz, 1H), 7.04 (t, *J* = 7.5 Hz, 1H), 5.33 (s, 2H). ^13^C NMR (151 MHz, CDCl_3_): δ 165.3, 157.2, 152.9, 152.5, 137.0, 135.0, 134.0, 131.4, 128.4, 123.5, 123.0, 122.6, 118.2, 113.1, 71.6; HRMS-ESI for [M + H]^+^ C_20_H_16_N_2_O_3_ calculated 355.1053; found, 355.1055.

#### 4.8.3 (*E*)-*N*-(3-(3,5-Bis(trifluoromethyl)phenyl)-1*H*-indol-7-yl)-2-((4-(phenyldiazenyl)benzyl)oxy)benzamide, C_36_H_24_F_6_N_4_O_2_ (**1b**)

In a 25 mL round-bottom flask, (*E*)-2-((4-(phenyldiazenyl)benzyl)oxy)benzoic acid **14** (200 mg, 0.60 mmol, 1 equiv) was dissolved in dry dichloromethane (30 mL). To this mixture were added thionyl chloride (1 mL) and a few drops of dry DMF and the reaction was refluxed at 70 °C for 5 h. The solvent was then evaporated using a rotary evaporator to give the crude mixture which was further concentrated twice with dry dichloromethane to remove excess of thionyl chloride. The red precipitate obtained was redissolved in dry dichloromethane and added slowly to the solution of **7a** (207 mg, 0.60 mmol, 1 equiv) in dry dichloromethane. DIPEA (100 μL, 0.60 mmol, 1 equiv) was added and the reaction mixture was allowed to stir at rt for further 3 h. After the completion of the reaction, the solvent was evaporated through a rotary evaporator, and the crude mixture was extracted with ethyl acetate (3 × 30 mL). The organic layers were dried with anhydrous magnesium sulfate, filtered, and concentrated under reduced pressure to give the crude product. The crude residue was then purified by column chromatography over silica gel to furnish **1b** as an orange solid (277 mg, 70%). ^1^H NMR (400 MHz, CDCl_3_): δ 10.77 (s, 1H), 10.37 (s, 1H), 8.40 (dd, *J* = 7.8, 1.8 Hz, 1H), 8.06–8.01 (m, 4H), 7.99–7.94 (m, 2H), 7.71 (d, *J* = 8.6 Hz, 3H), 7.63–7.51 (m, 5H), 7.48 (d, *J* = 2.6 Hz, 1H), 7.25–7.20 (m, 2H), 6.92 (t, *J* = 7.8 Hz, 1H), 6.25 (d, *J* = 7.6 Hz, 1H), 5.38 (s, 2H); ^13^C NMR (151 MHz, CDCl_3_): δ 162.9, 156.7, 153.2, 152.5, 138.0, 137.4, 133.8, 132.8, 132.2–131.6 (q), 131.5, 129.3, 129.1, 128.8, 127.7, 127.05, 126.3–120.8 (q), 123.7, 123.6, 123.5, 123.0, 122.3, 121.1, 120.7, 119.09–118.9 (q), 115.8, 115.3, 113.9, 112.8, 71.6; HRMS-ESI for [M + H]^+^ C_36_H_24_F_6_N_4_O_2_ calculated 681.1696; found, 681.1700.

## Supporting Information Available

Anion binding data, ion transport procedures and data, and NMR spectra (PDF)
