# Supplementary material for: Off–On Photo- and Redox-Triggered Anion Transport Using an Indole-Based Hydrogen Bond Switch
Source: ACS Omega. 2024 Nov 1;9(45):45572–80. doi: 10.1021/acsomega.4c07880 (PMC11561614; doi:10.1021/acsomega.4c07880)
Supplement: Supplementary file 1 — ao4c07880_si_001.pdf [file ao4c07880_si_001.pdf]

Supporting Information for

Off-on photo- and redox-triggered anion transport using an indole-based hydrogen bond switch

Manzoor Ahmad[a], Andrew Muir[a], and Matthew J. Langton*[a]

Contents

Contents 1
I. NMR spectra 2
II. Anion Binding Studies 19
III. Ion transport studies 24
IV. Stimulus-responsive triggered activation in the solution phase 30
V. Stimulus-responsive ion transport activation 33
VI. References 35

I. NMR spectra

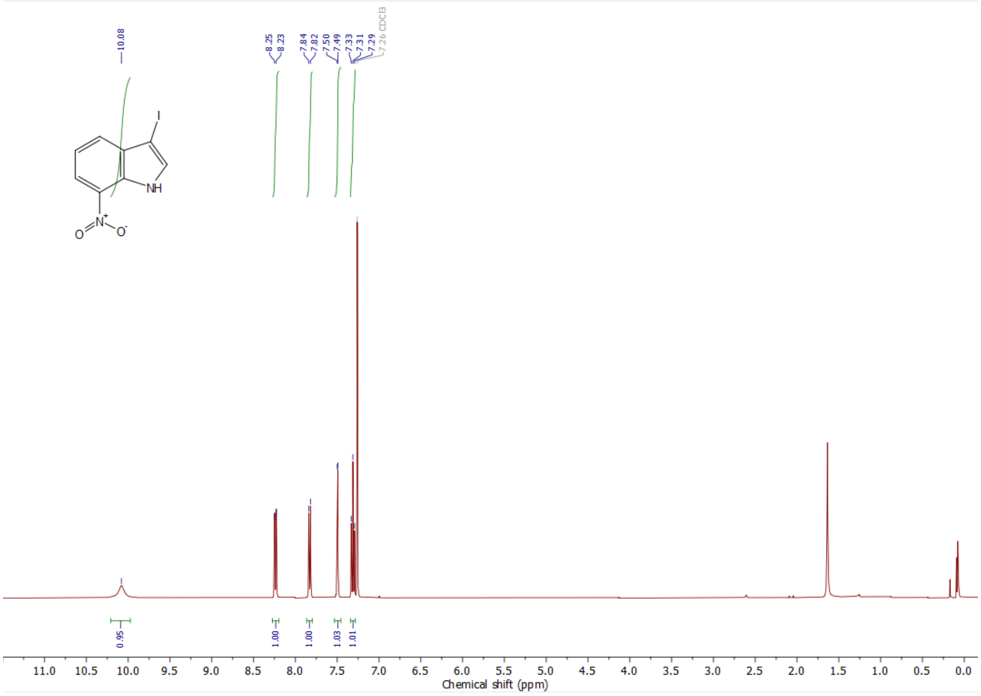

Figure S1: ^{1}H NMR Spectrum of **5** in $CDCl_3$, 298 K.

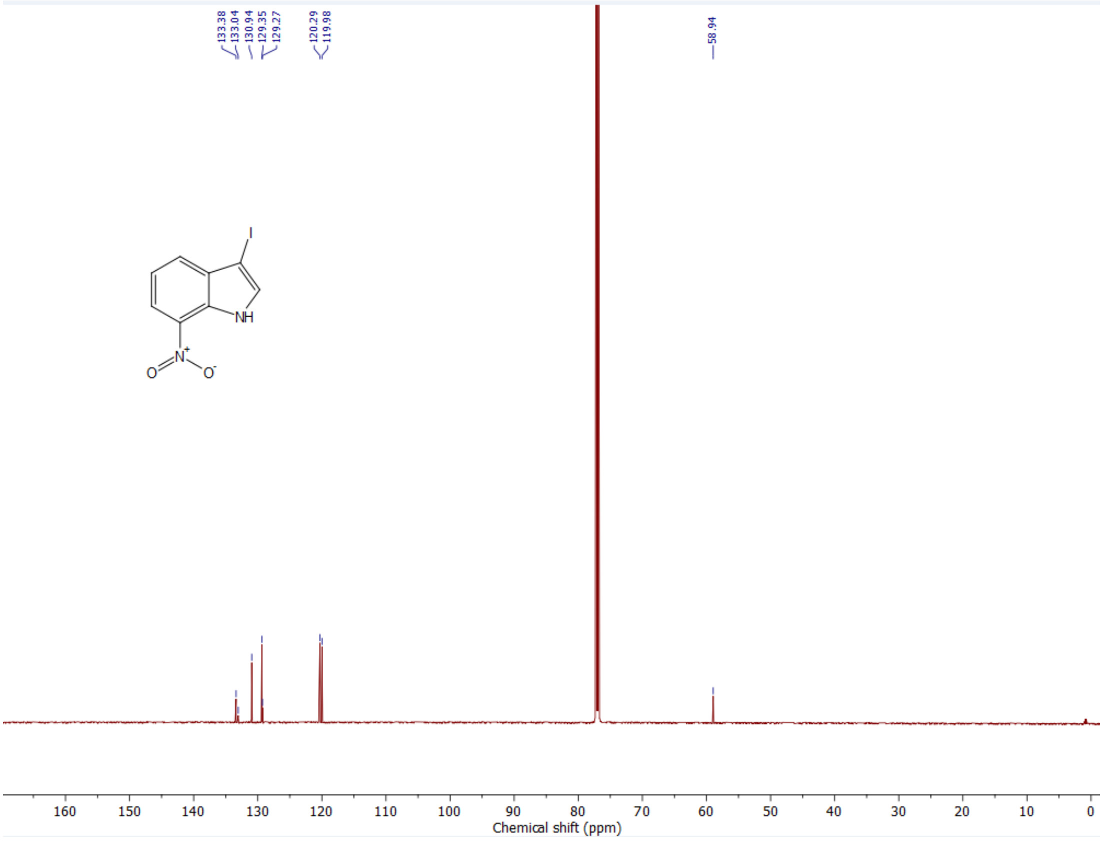

Figure S2: ^{13}C NMR Spectrum of **5** in $CDCl_3$, 298 K.

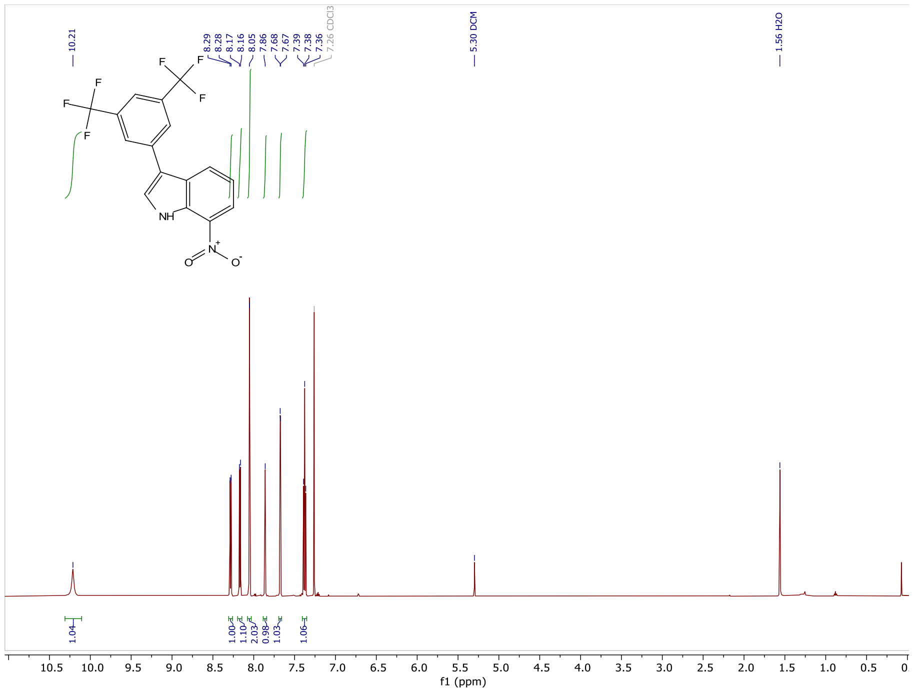

Figure S3: ^{1}H NMR Spectrum of **6a** in $CDCl_3$, 298 K.

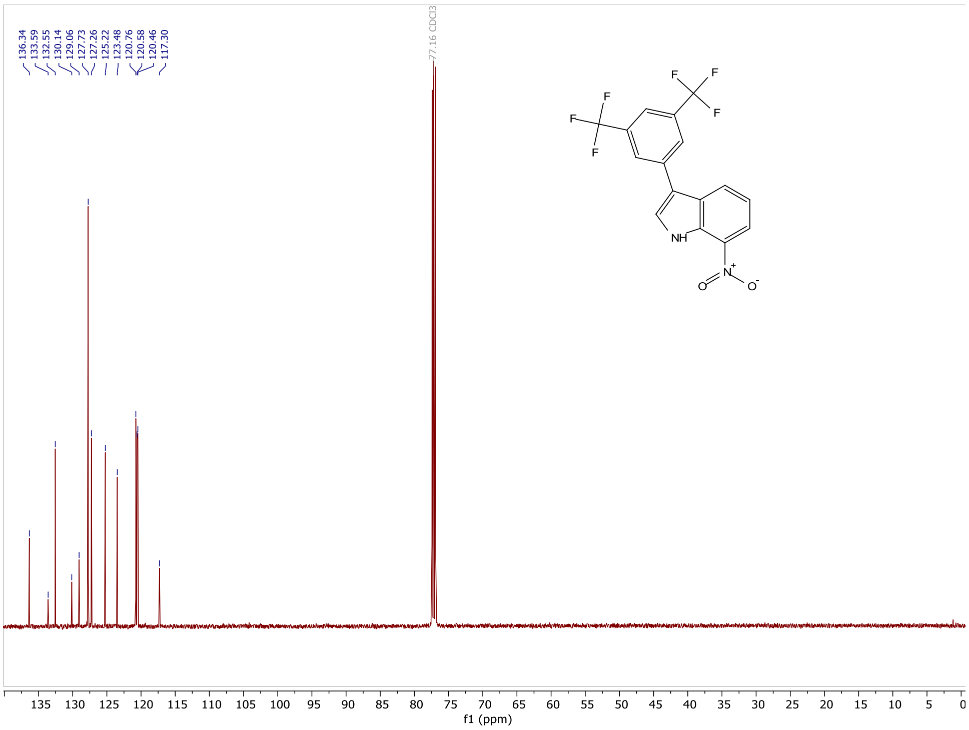

Figure S4: ^{13}C NMR Spectrum of **6a** in $CDCl_3$, 298 K.

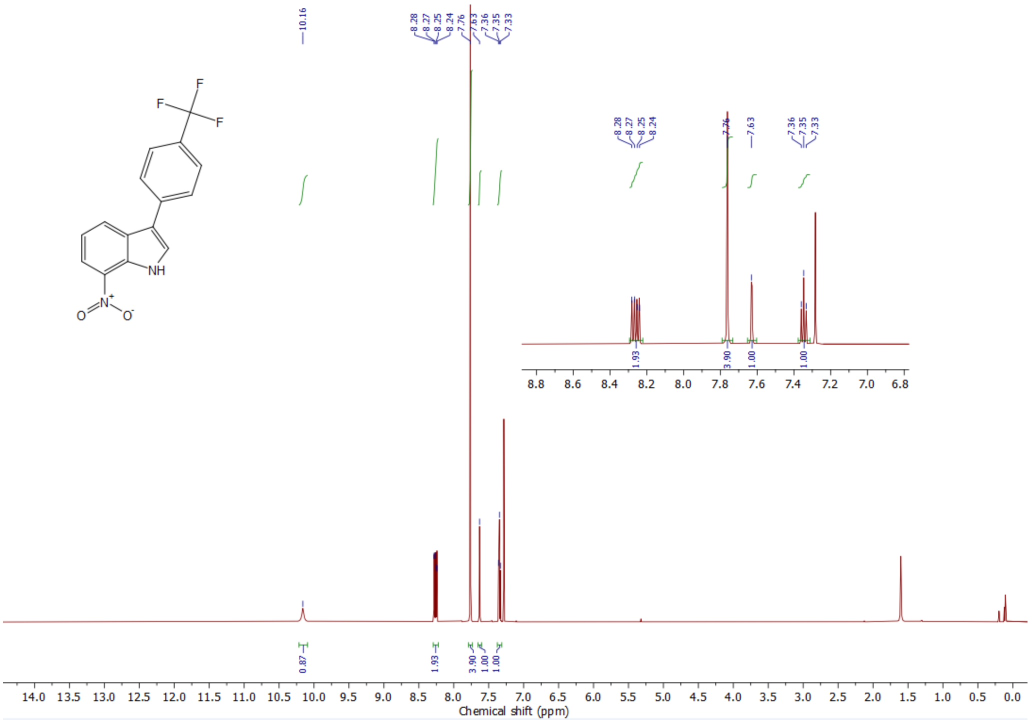

Figure S5: ^{1}H NMR Spectrum of **6b** in $CDCl_3$, 298 K.

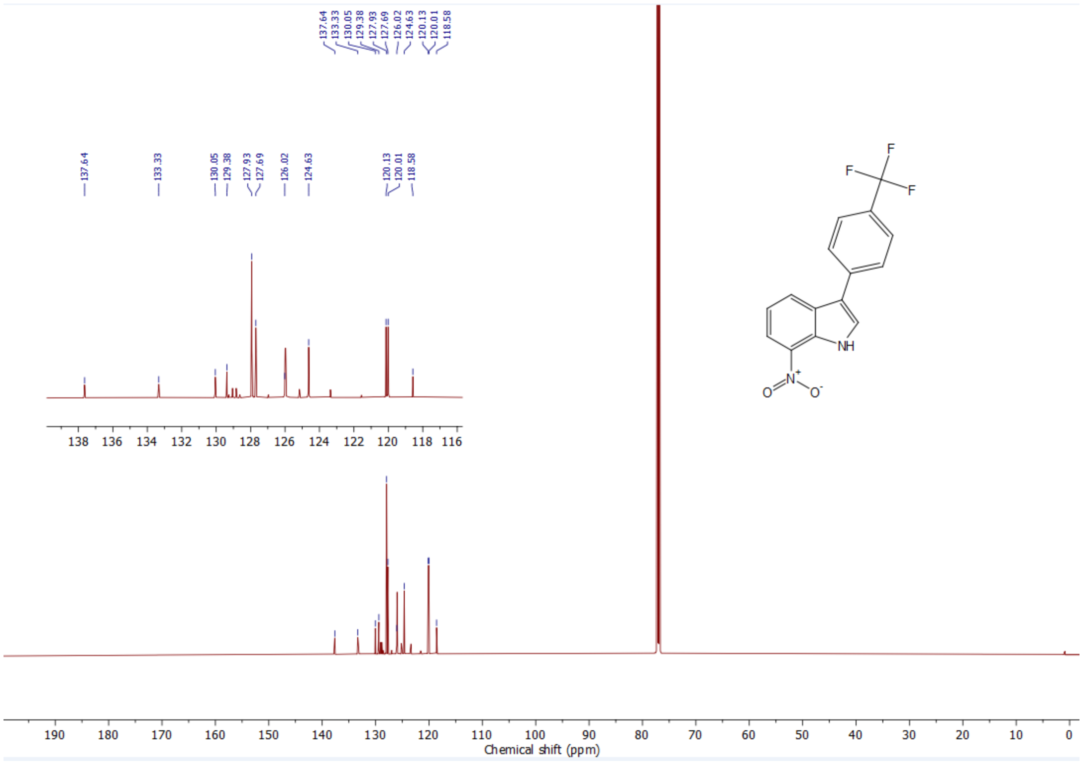

Figure S6: ^{13}C NMR Spectrum of **6b** in $CDCl_3$, 298 K.

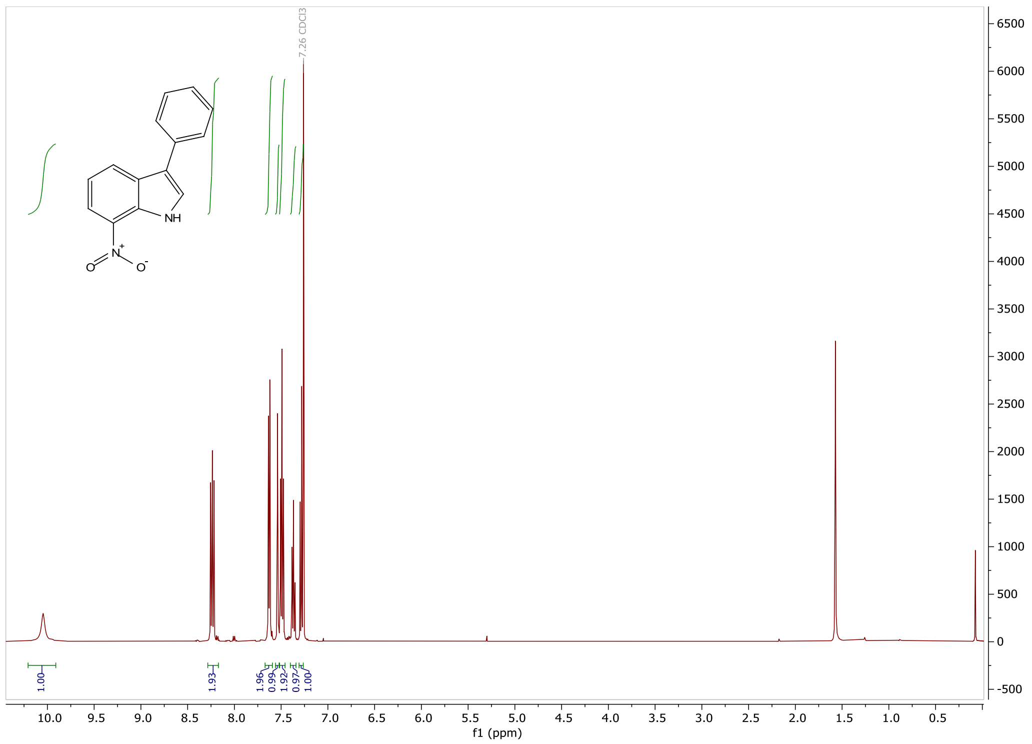

Figure S7: ^{1}H NMR Spectrum of **6c** in $CDCl_3$, 298 K.

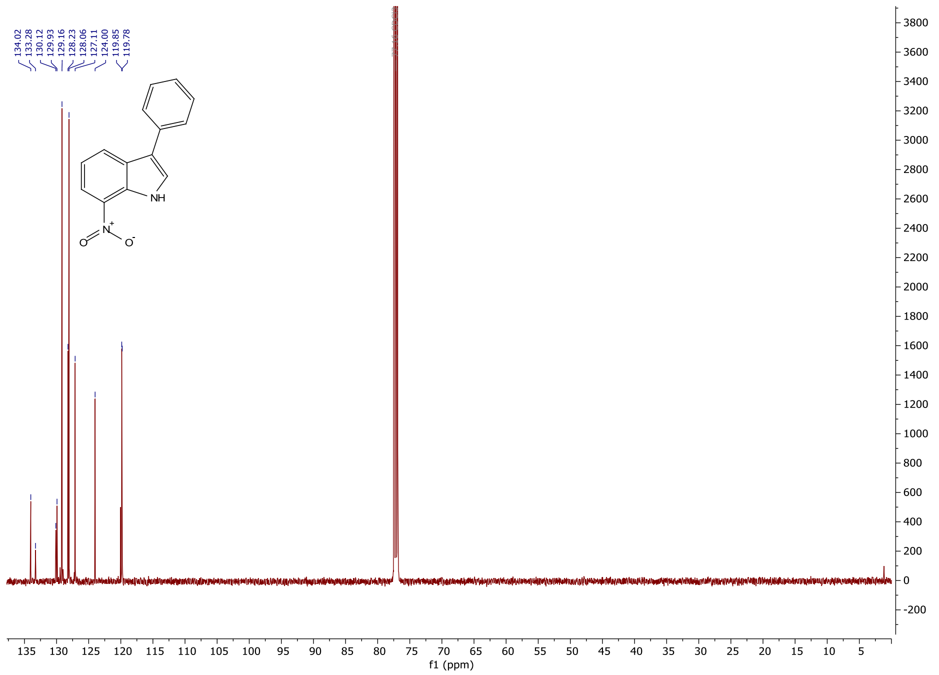

Figure S8: ^{13}C NMR Spectrum of **6c** in $CDCl_3$, 298 K.

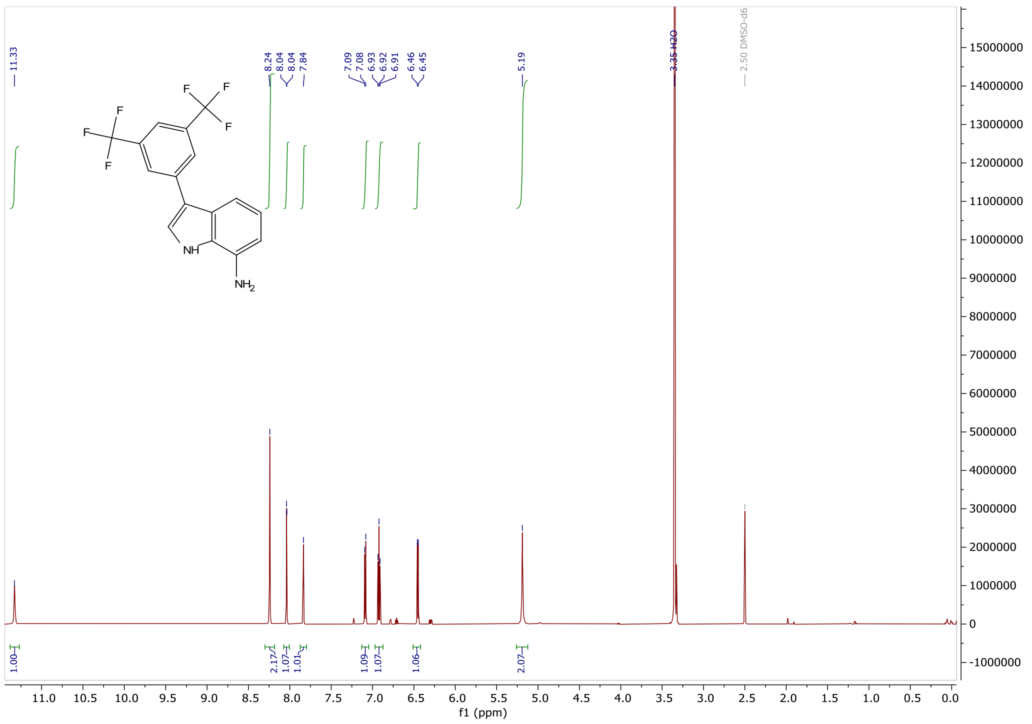

Figure S9: ^{1}H NMR Spectrum of **7a** in DMSO-d_6, 298 K.

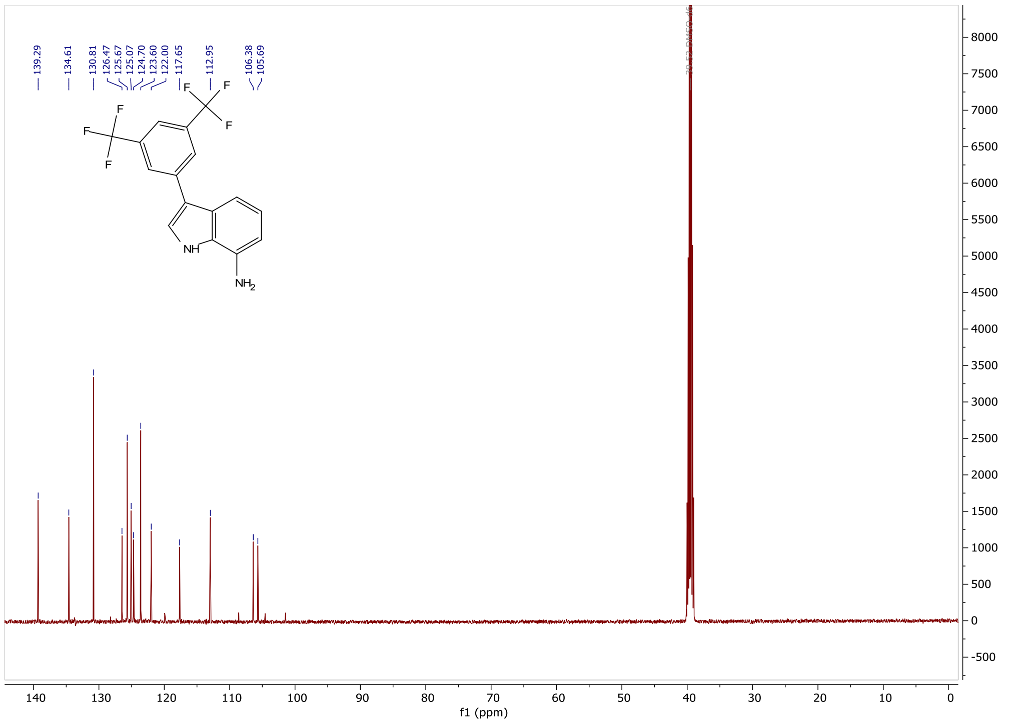

Figure S10: ^{13}C NMR Spectrum of **7a** in DMSO-d_6, 298 K.

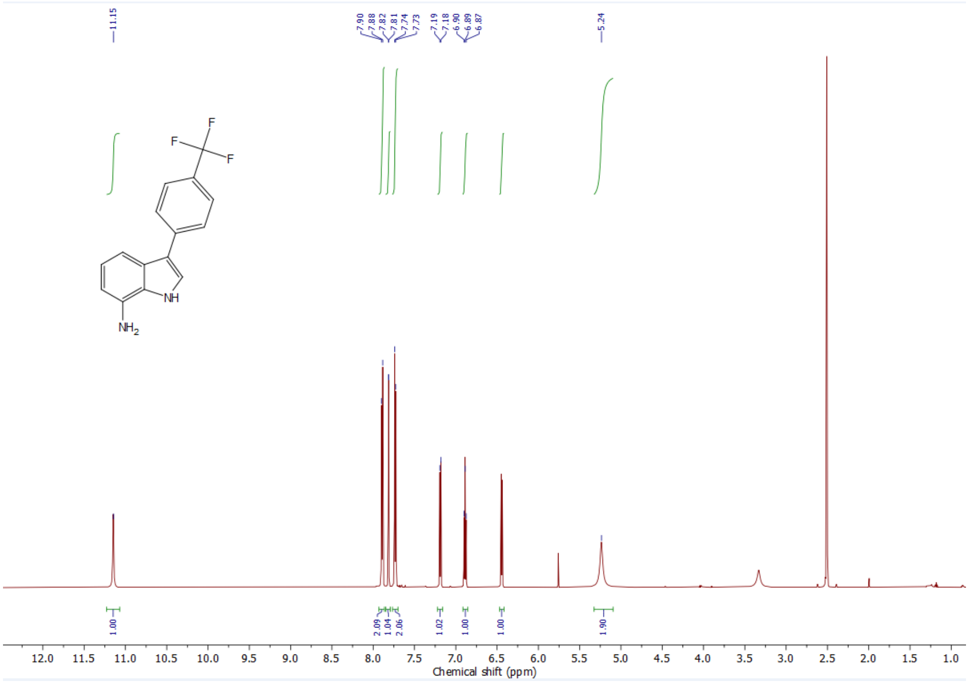

Figure S11: ^{1}H NMR Spectrum of **7b** in DMSO-d_6, 298 K.

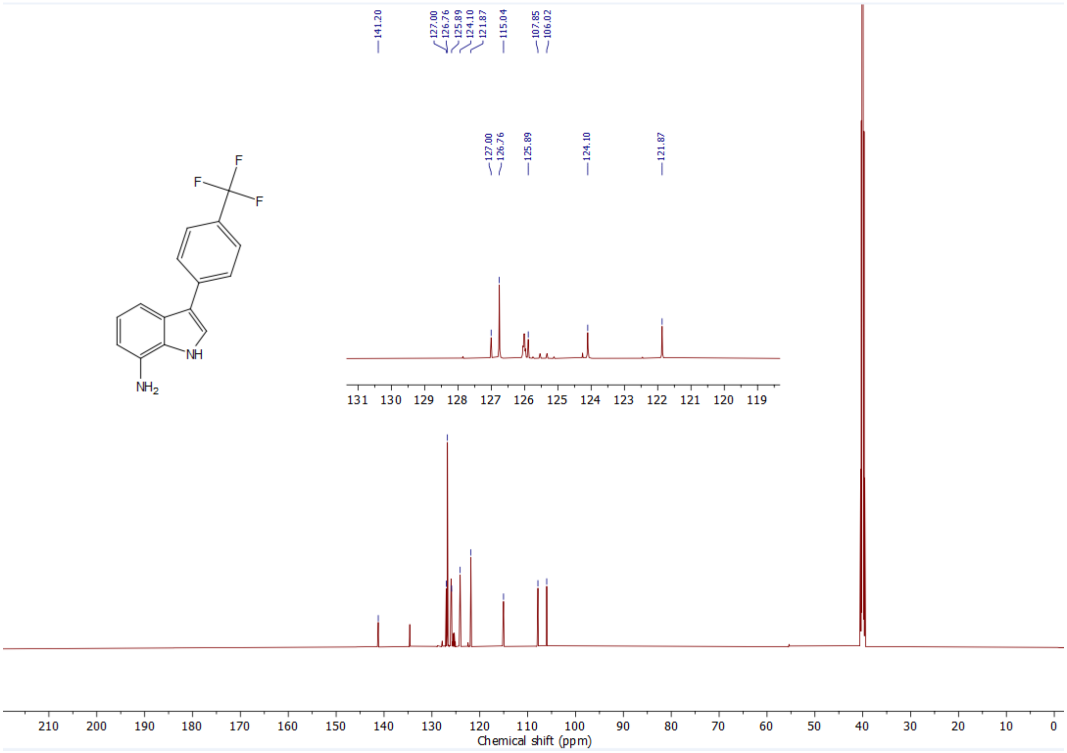

Figure S12: ^{13}C NMR Spectrum of **7b** in DMSO-d_6, 298 K.

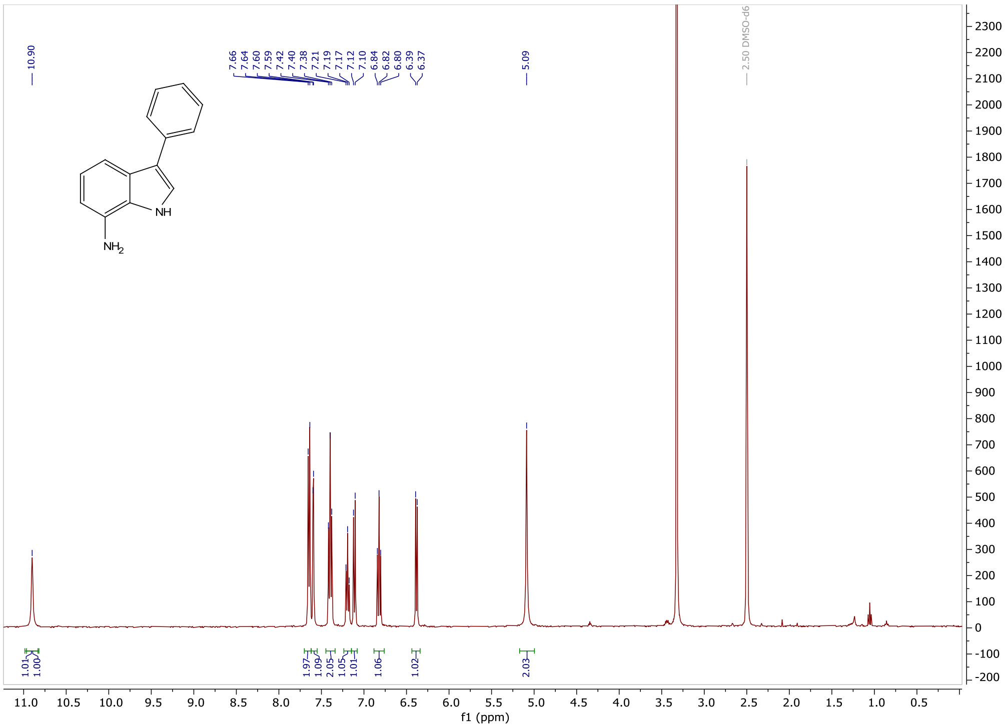

Figure S13: ^{1}H NMR Spectrum of **7c** in DMSO-d_6, 298 K.

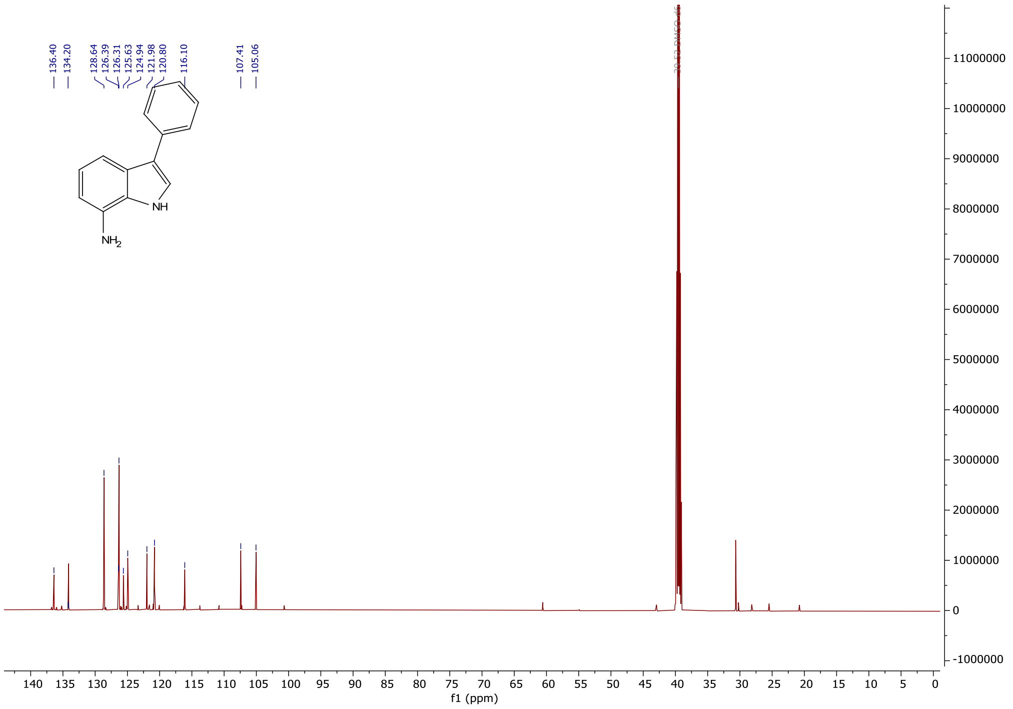

Figure S14: ^{13}C NMR Spectrum of **7c** in DMSO-d_6, 298 K.

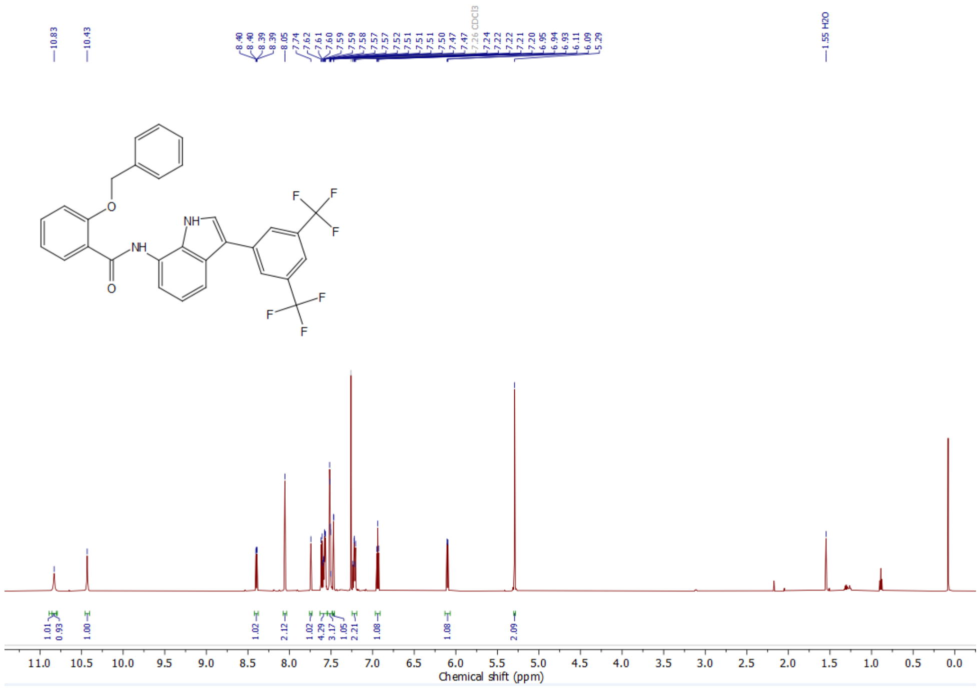

Figure S15: ^{1}H NMR Spectrum of **9a** in $CDCl_3$, 298 K.

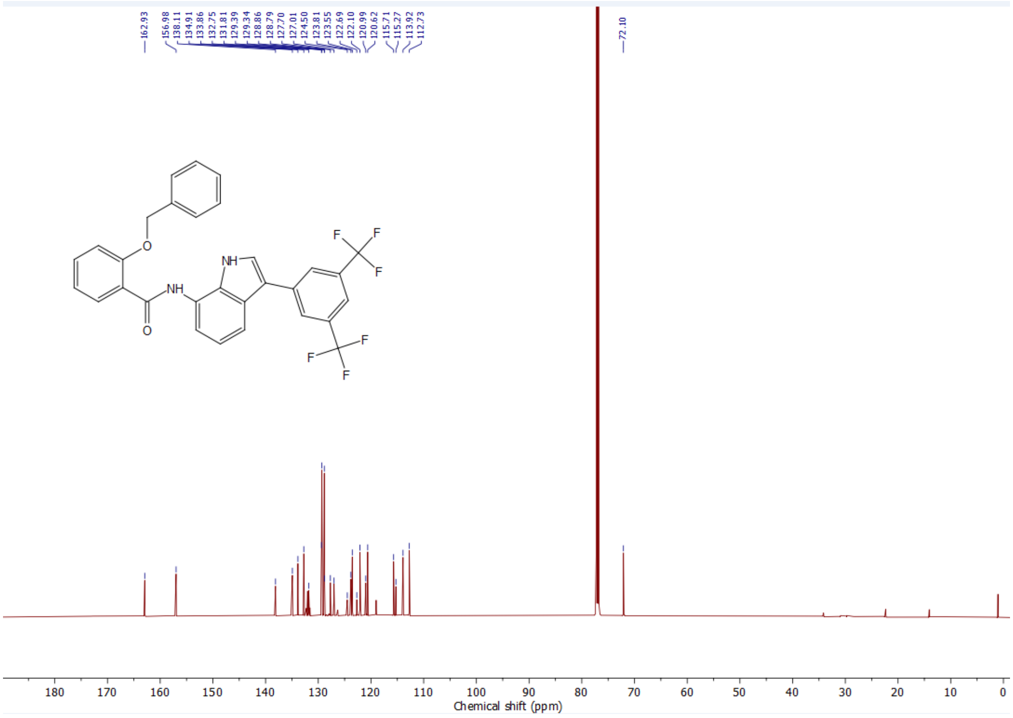

Figure S16: ^{13}C NMR Spectrum of **9a** in $CDCl_3$, 298 K.

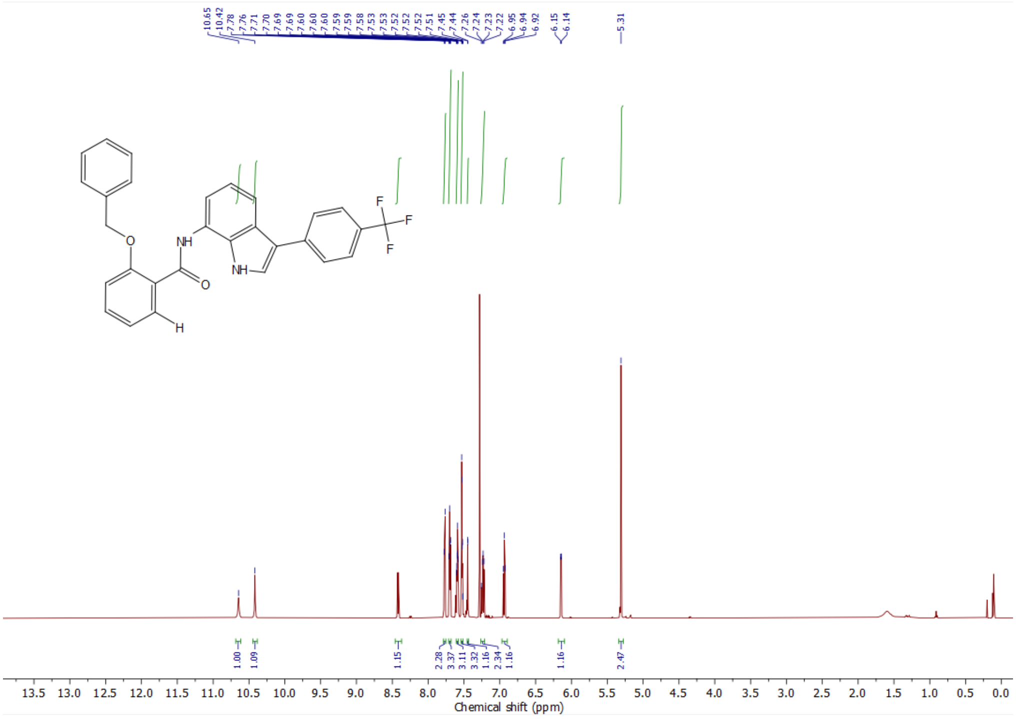

Figure S17: ^{1}H NMR Spectrum of **9b** in $CDCl_3$, 298 K.

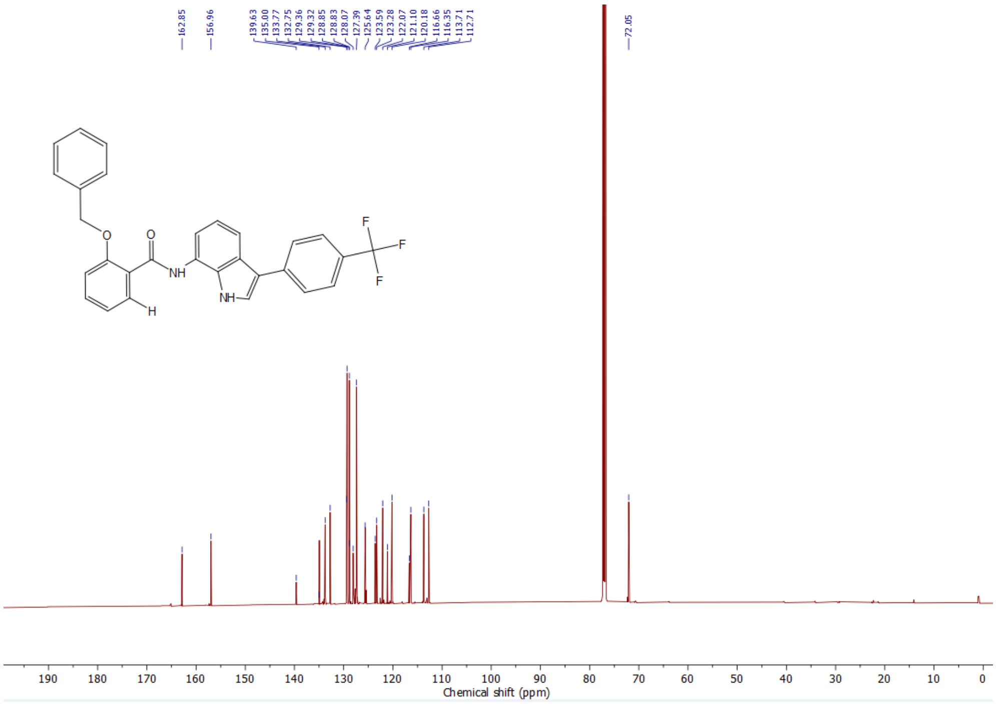

Figure S18: ^{13}C NMR Spectrum of **9b** in $CDCl_3$, 298 K.

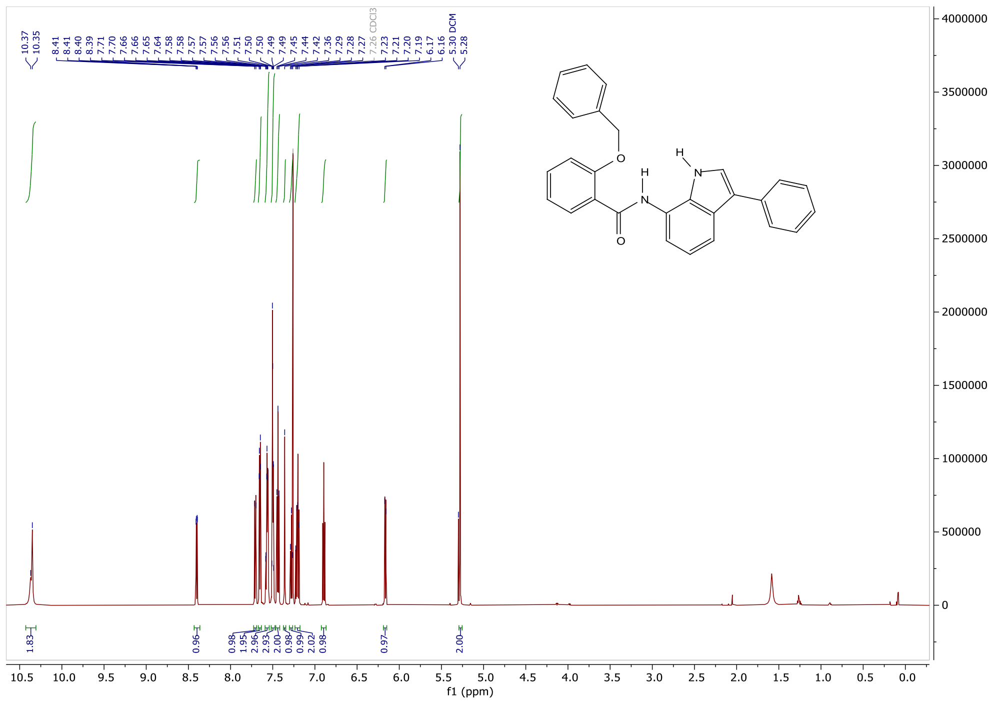

Figure S19: ^{1}H NMR Spectrum of **9c** in $CDCl_3$, 298 K.

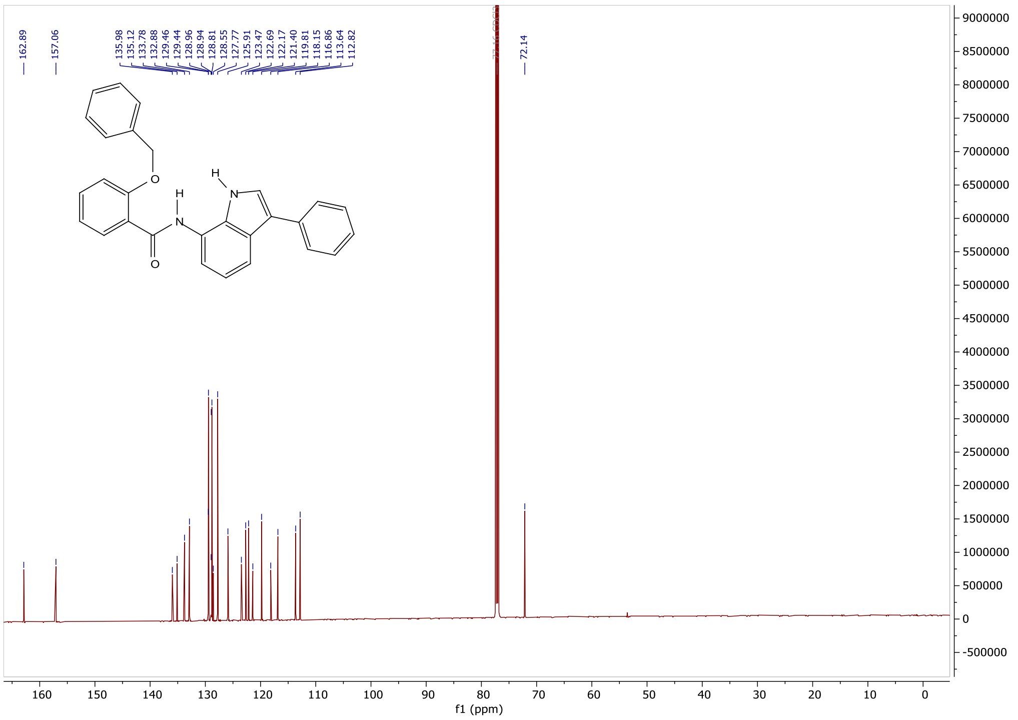

Figure S20: ^{13}C NMR Spectrum of **9c** in $CDCl_3$, 298 K.

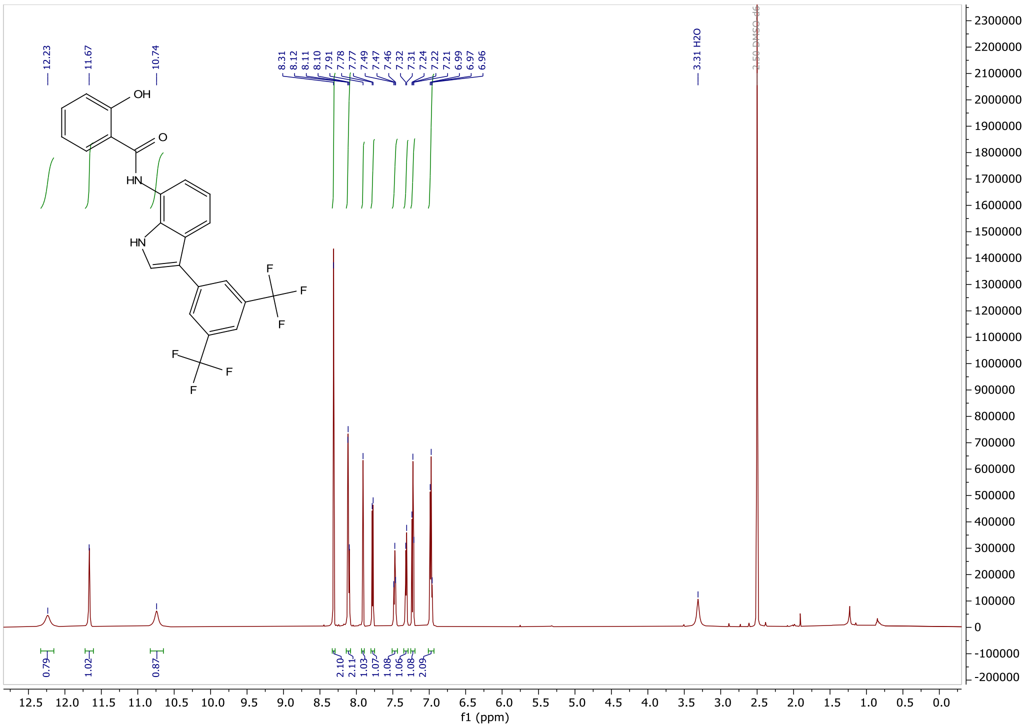

Figure S21: ^{1}H NMR Spectrum of **1** in DMSO-d_6, 298 K.

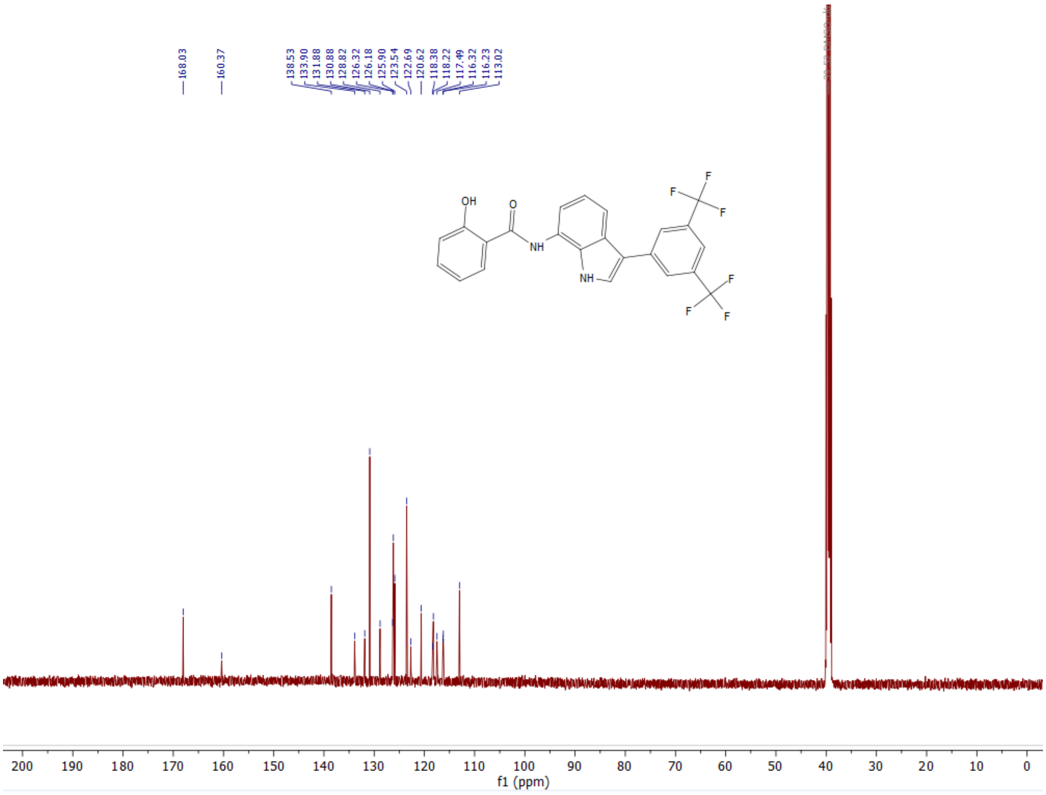

Figure S22: ^{13}C NMR Spectrum of **1** in DMSO-d_6, 298 K.

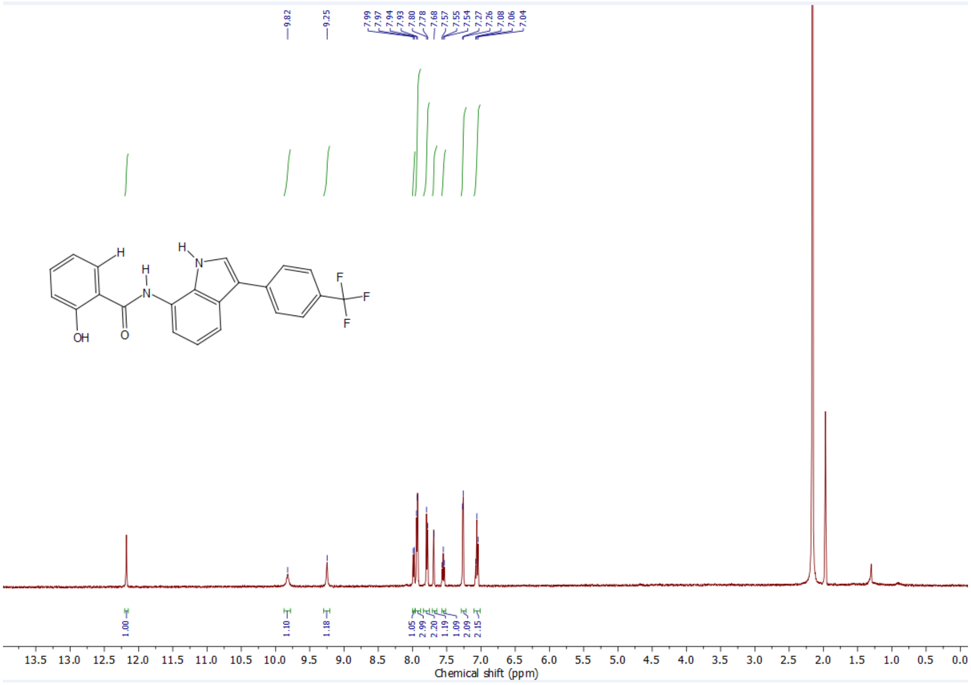

Figure S23: ^{1}H NMR Spectrum of **2** in acetonitrile-d_3, 298 K.

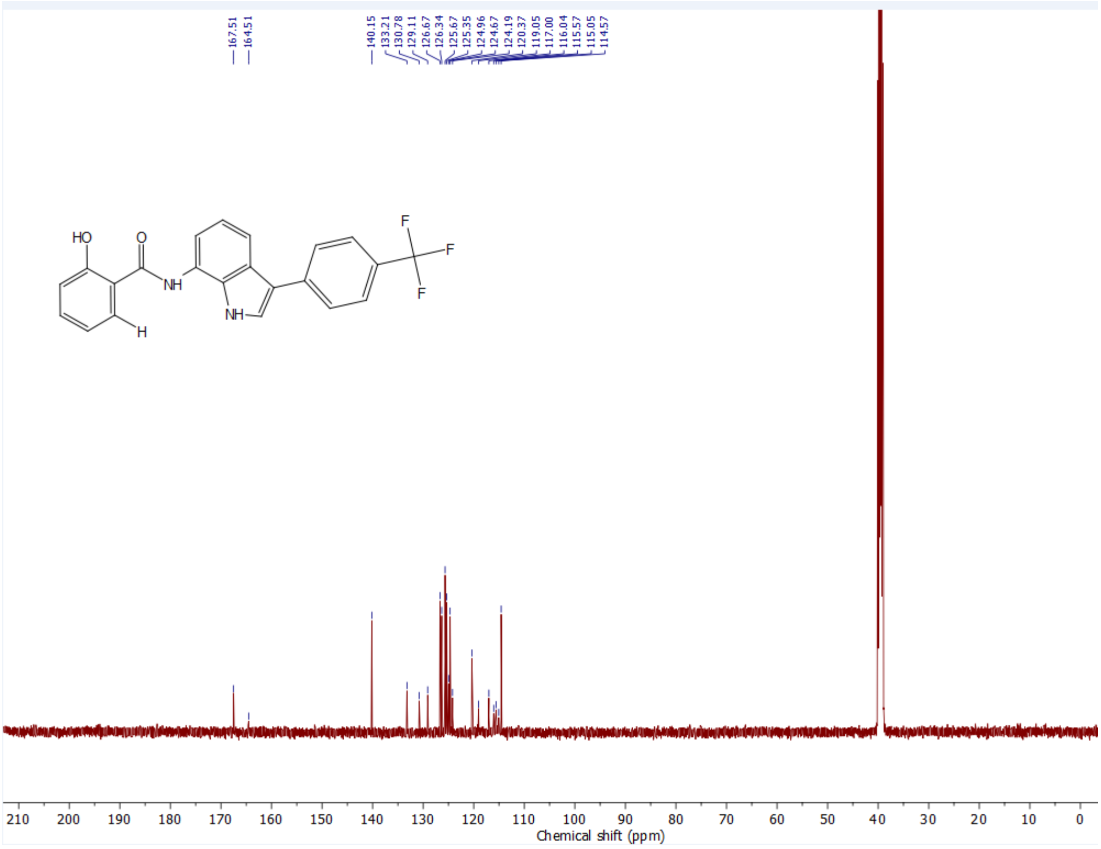

Figure S24: ^{13}C NMR Spectrum of **2** in DMSO-d_6, 298 K.

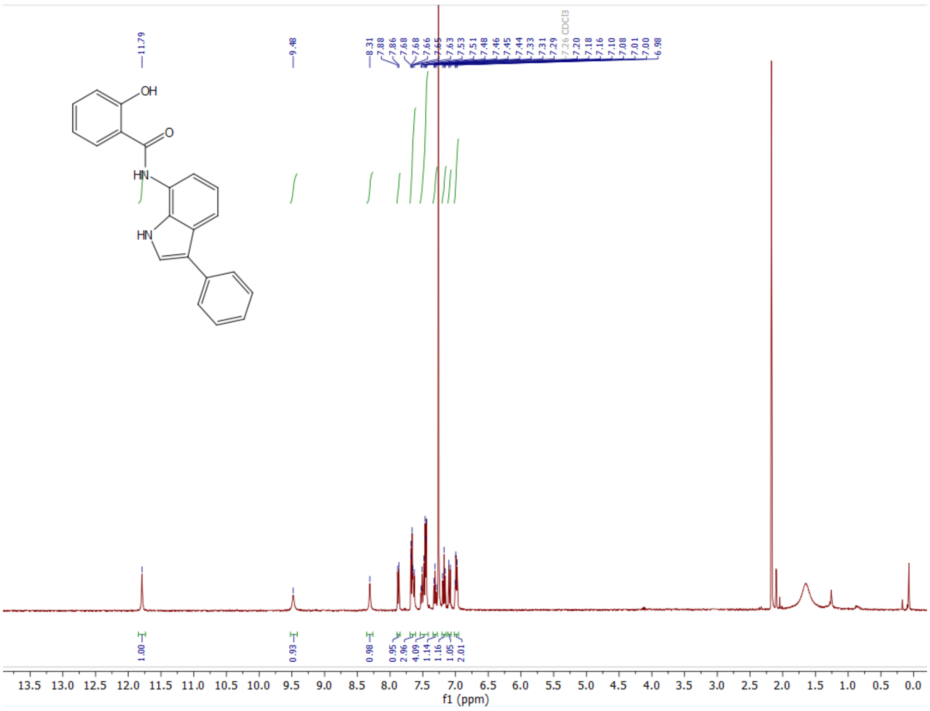

Figure S25: ^{1}H NMR Spectrum of **3** in $CDCl_3$, 298 K.

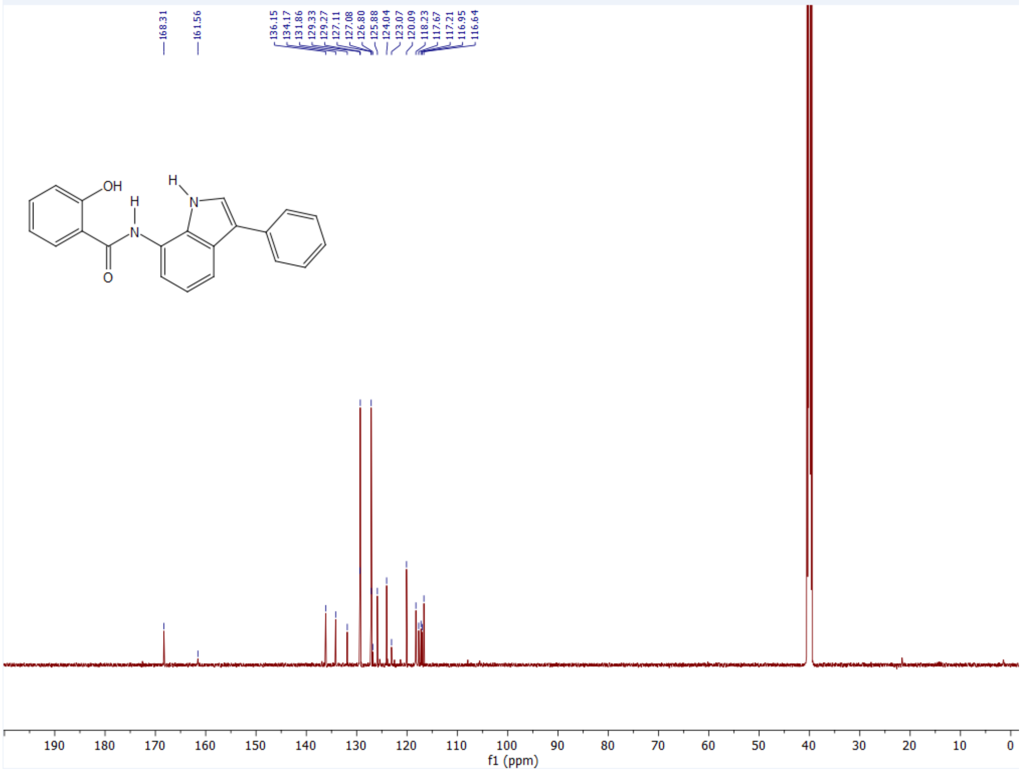

Figure S26: ^{13}C NMR Spectrum of **3** in $CDCl_3$, 298 K.

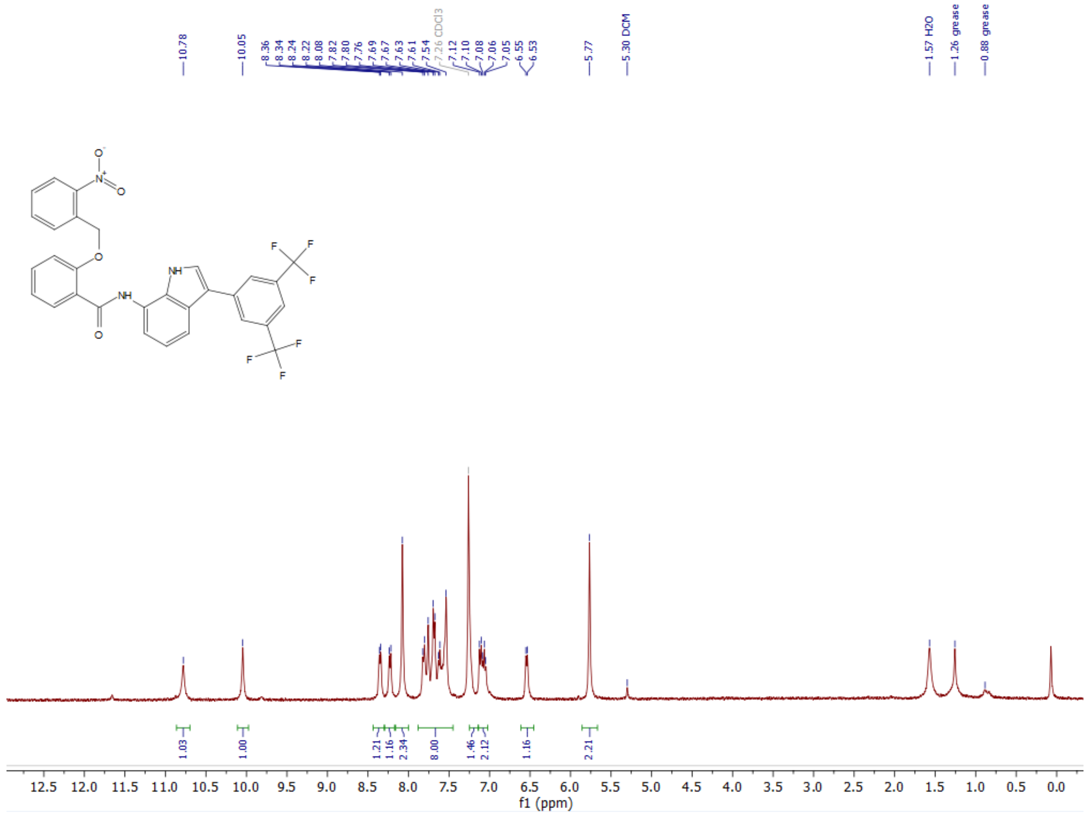

Figure S27: ^{1}H NMR Spectrum of **1a** in $CDCl_3$, 298 K.

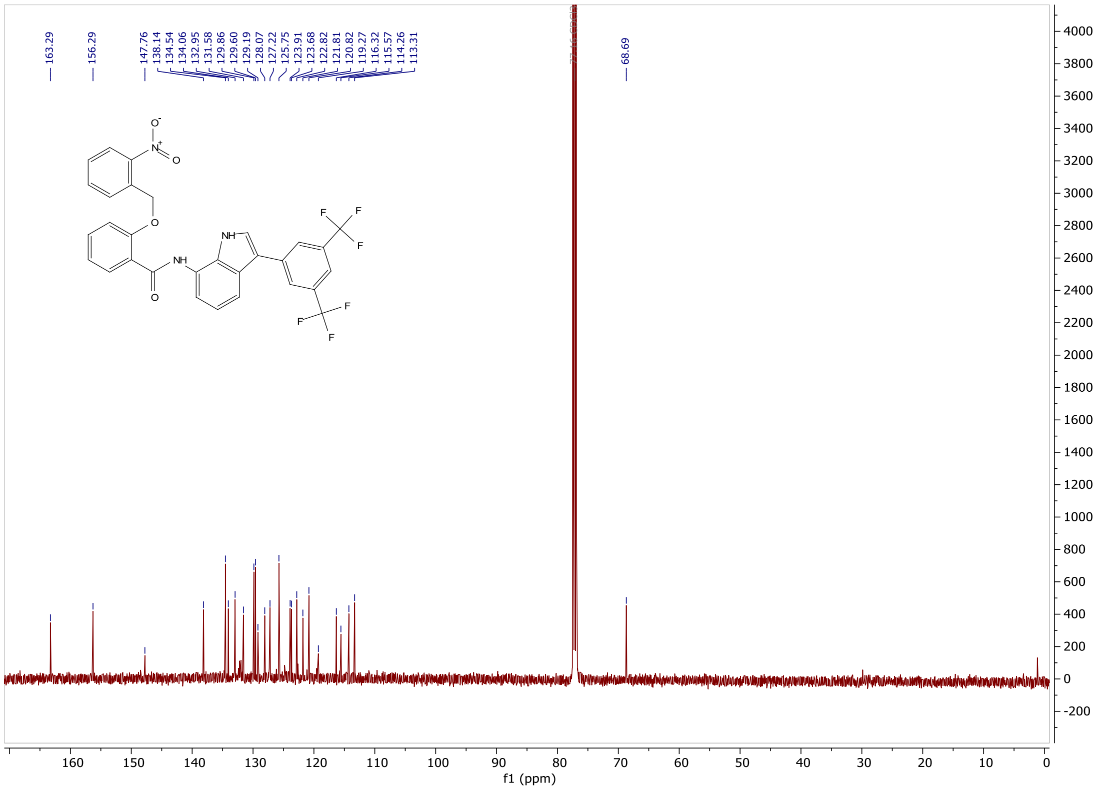

Figure S28: ^{13}C NMR Spectrum of **1a** in $CDCl_3$, 298 K.

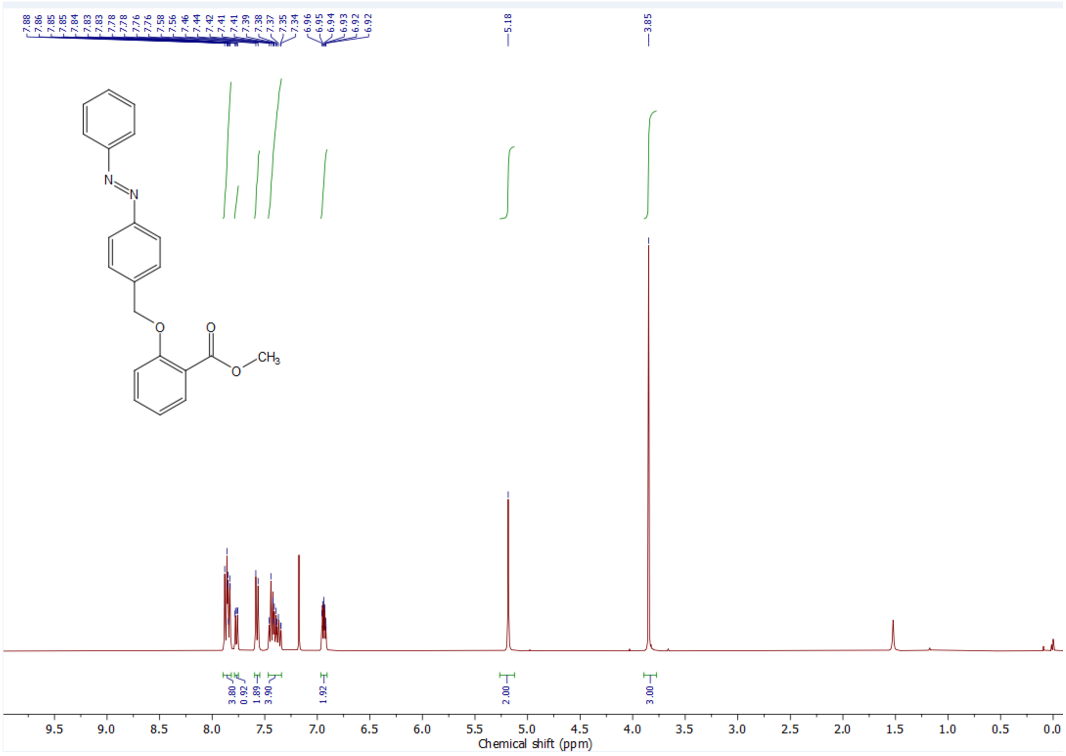

Figure S29: ^{1}H NMR Spectrum of **13** in $CDCl_3$, 298 K.

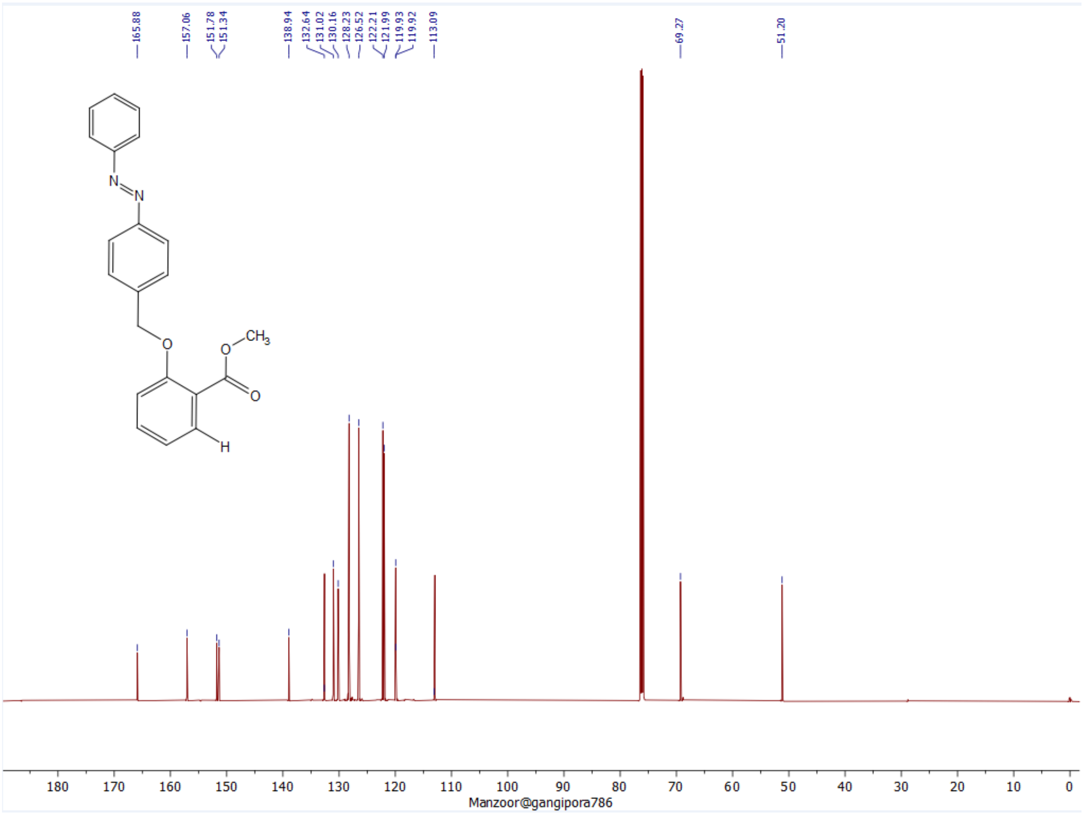

Figure S30: ^{13}C NMR Spectrum of **13** in $CDCl_3$, 298 K.

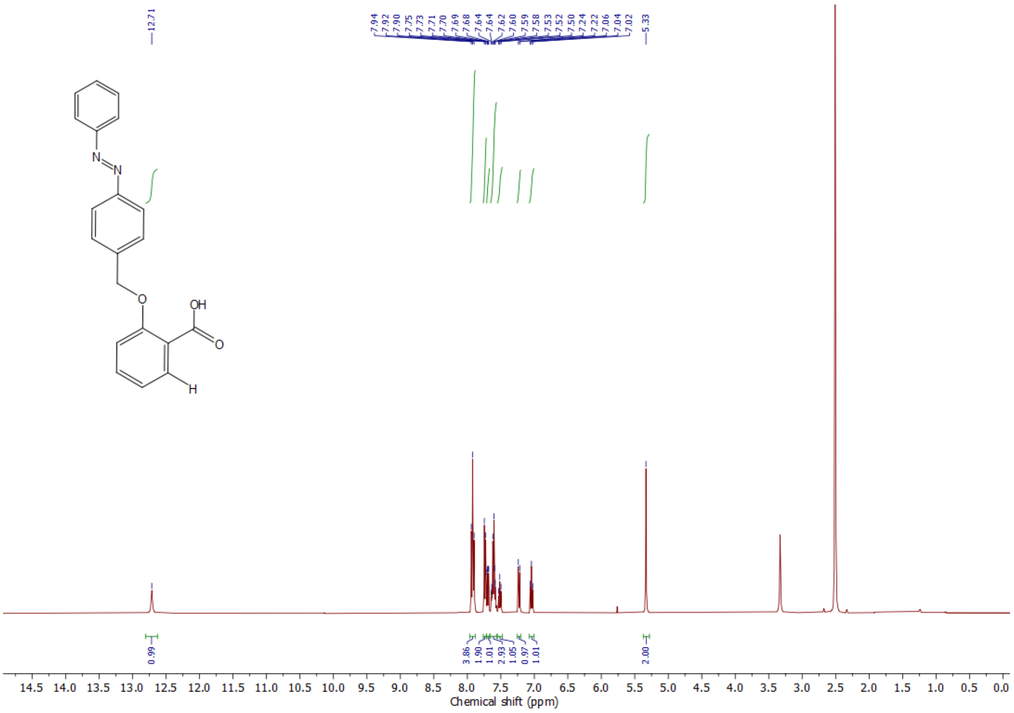

Figure S31: ^{1}H NMR Spectrum of **14** in DMSO-d_6, 298 K.

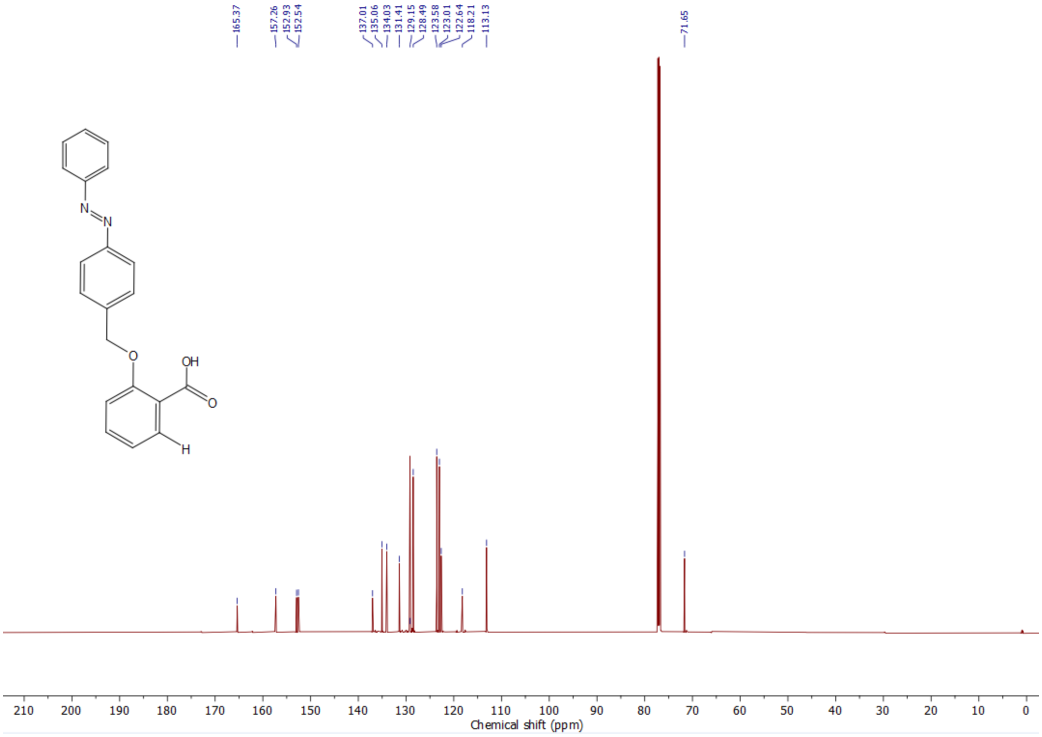

Figure S32: ^{13}C NMR Spectrum of **14** in DMSO-d_6, 298 K.

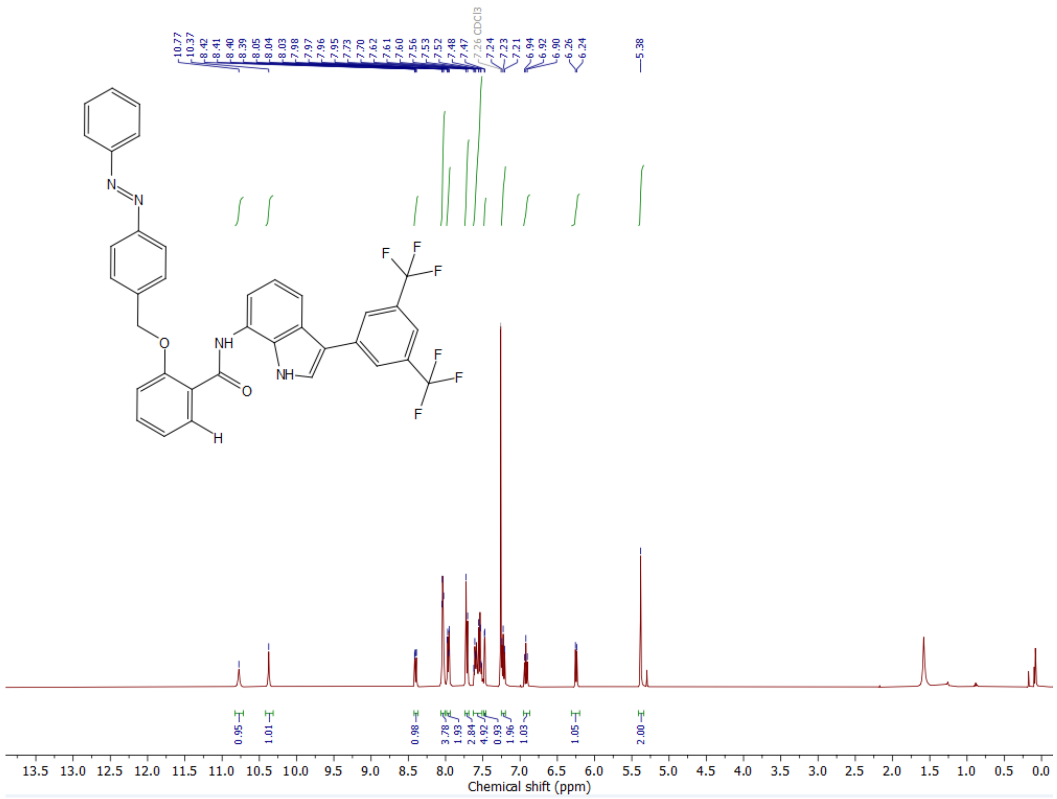

Figure S33: ^{1}H NMR Spectrum of **1b** in $CDCl_3$, 298 K.

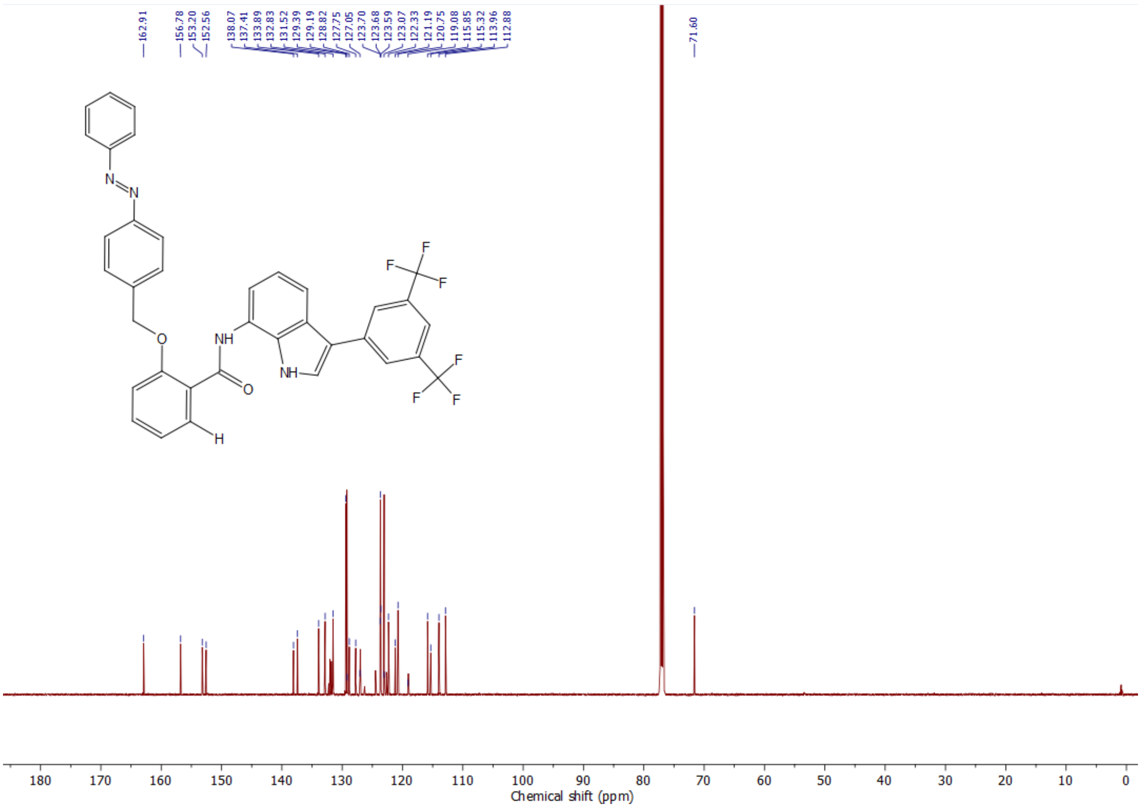

Figure S34: ^{13}C NMR Spectrum of **1b** in $CDCl_3$, 298 K.

II. Anion Binding Studies

Transporters 1-4:

^1H NMR titration experiments was carried out at room temperature on Bruker 400 MHz spectrometer. The residual solvent signal (CD_3CN, $\delta_H = 1.94$) was considered as an internal reference to calibrate spectra. TBACl and receptor were dried under a high vacuum before use. The titrations were performed by the addition of aliquots from the solution of TBACl (0.25 M in CD_3CN) to the solution of receptors either of **1**, **2**, **3**, **1a** or **1b** (2.5 mM), respectively. All NMR data were processed using MestReNova 6.0 and the collected data analysed using BindFit v0.5.[2]

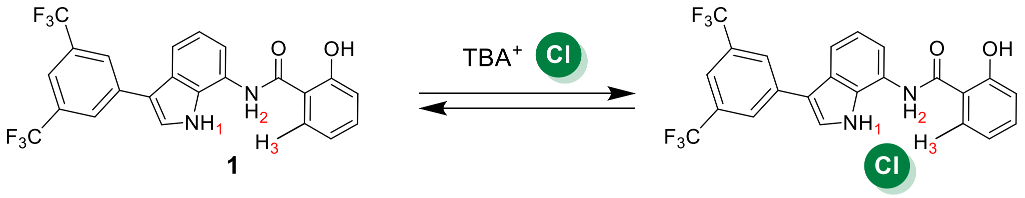

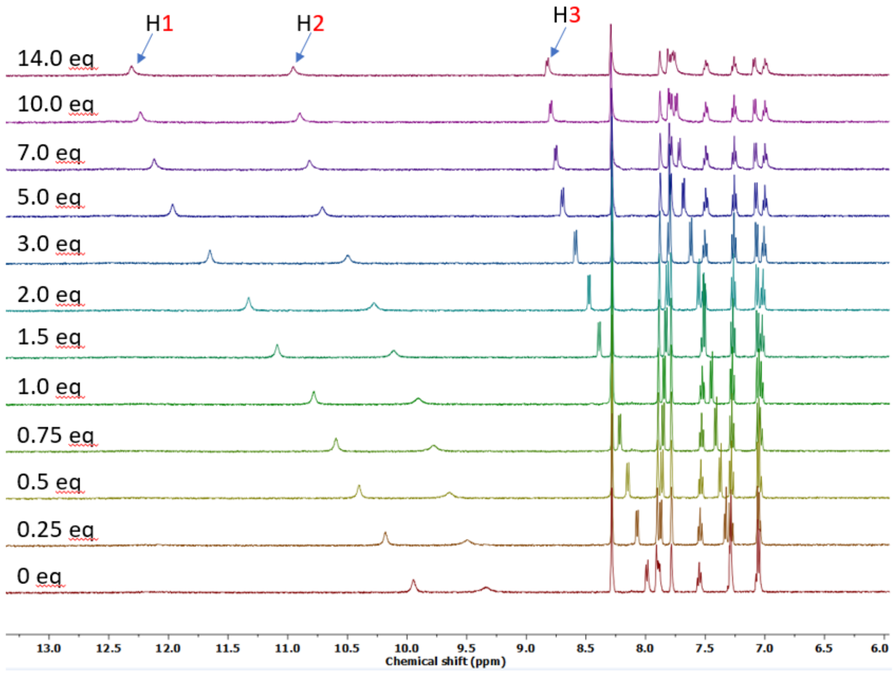

Figure S35. ^1H NMR titration spectra for **1** (2.5 mM in CD_3CN) with stepwise addition of TBACl in CD_3CN. The equivalents of added TBACl are shown on the stacked spectra.

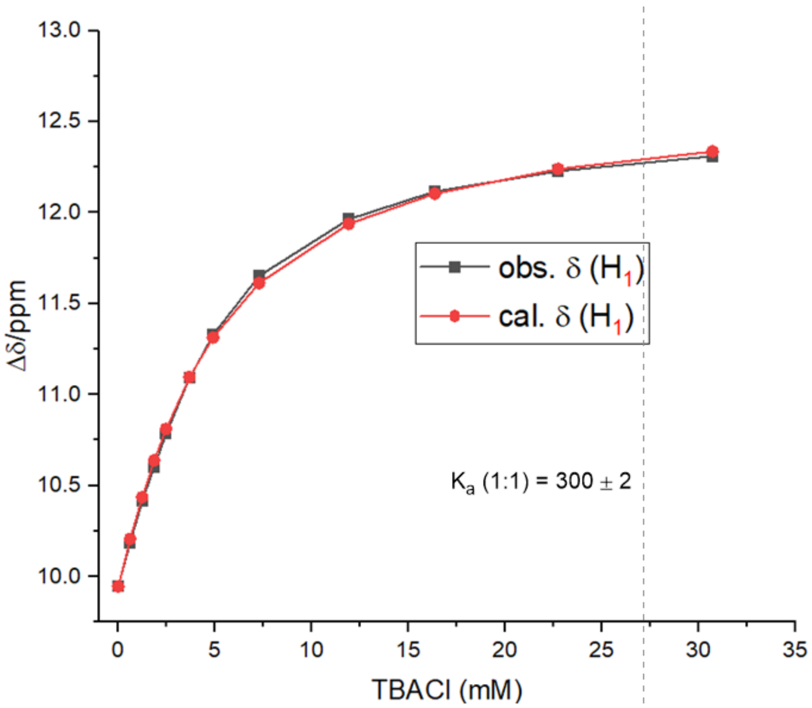

Figure S36. The plot of chemical shift (δ) of H_1 proton *vs* concentration of TBACl added, fitted to 1:1 binding model of BindFit v0.5.

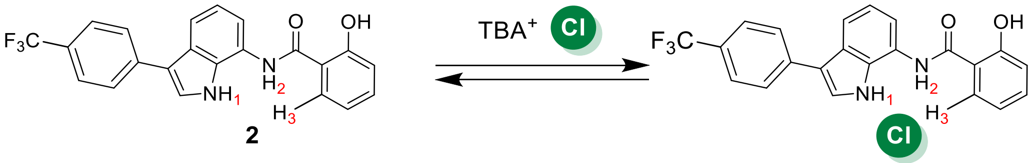

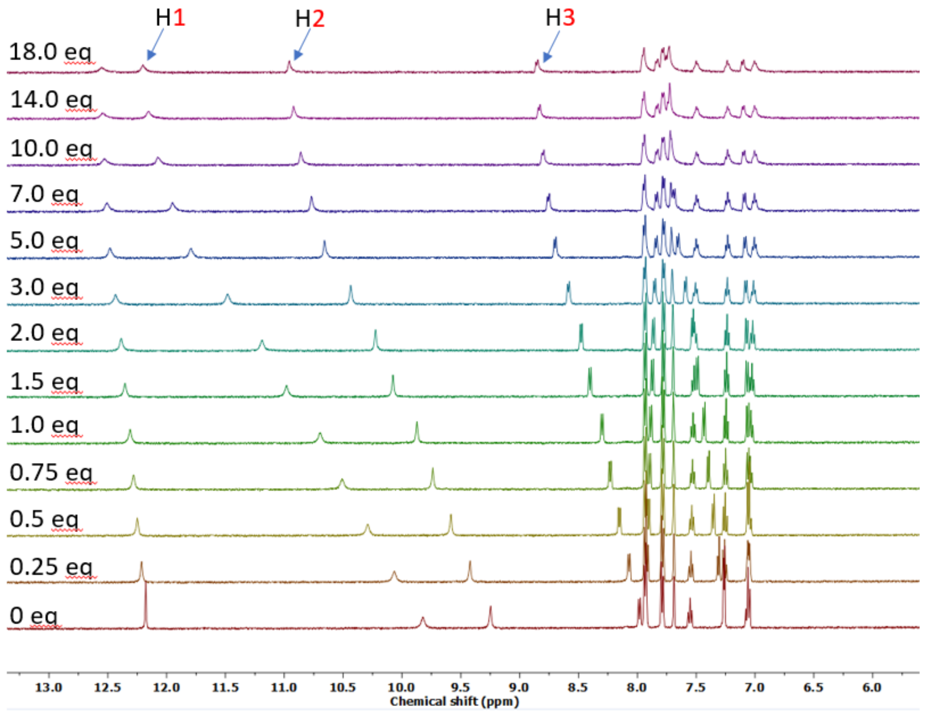

Figure S37. ^{1}H NMR titration spectra for **2** (2.5 mM in CD_3CN) with stepwise addition of TBACl in CD_3CN. The equivalents of added TBACl are shown on the stacked spectra.

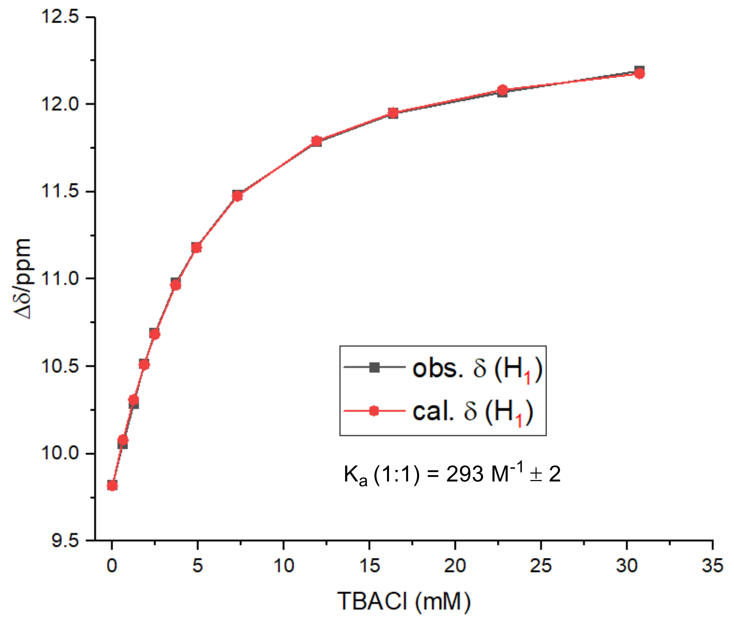

Figure S38. The plot of chemical shift (δ) of H_1 proton *vs* concentration of TBACl added, fitted to 1:1 binding model of BindFit v0.5.

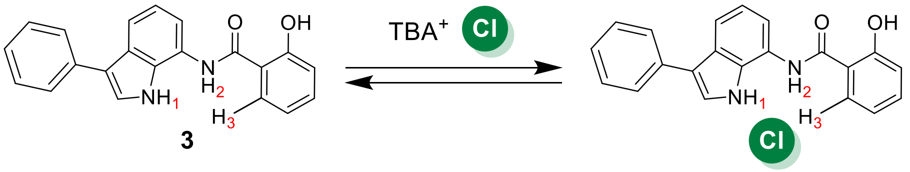

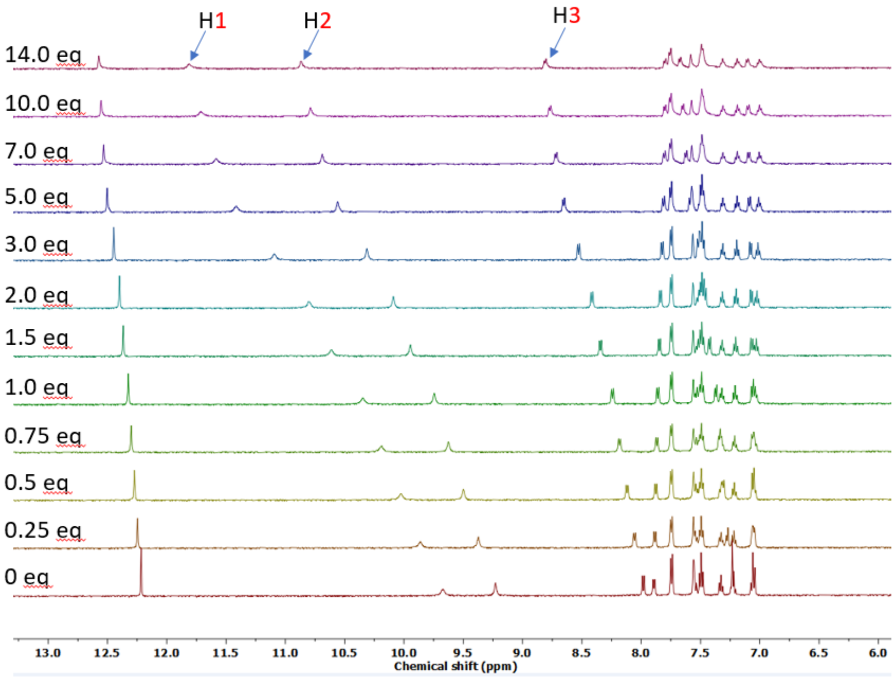

Figure S39. ^{1}H NMR titration spectra for **3** (2.5 mM in CD_3CN) with stepwise addition of TBACl in CD_3CN. The equivalents of added TBACl are shown on the stacked spectra.

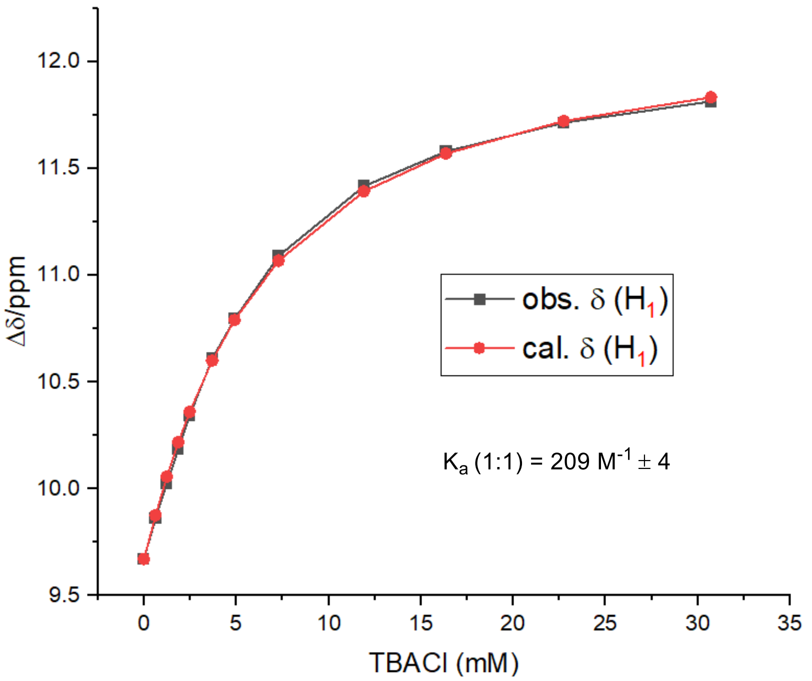

Figure S40. The plot of chemical shift (δ) of H_1 proton *vs* concentration of TBACl added, fitted to 1:1 binding model of BindFit v0.5.

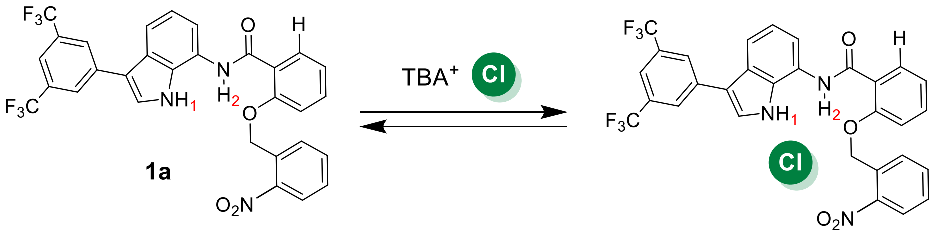

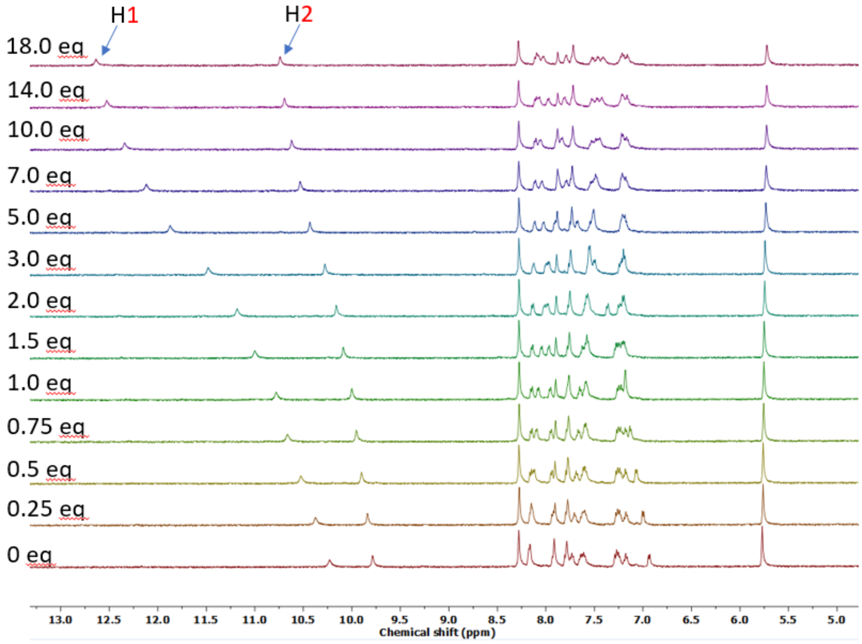

Figure S41. ^{1}H NMR titration spectra for **1a** (2.5 mM in CD_3CN) with stepwise addition of TBACl in CD_3CN. The equivalents of added TBACl are shown on the stacked spectra.

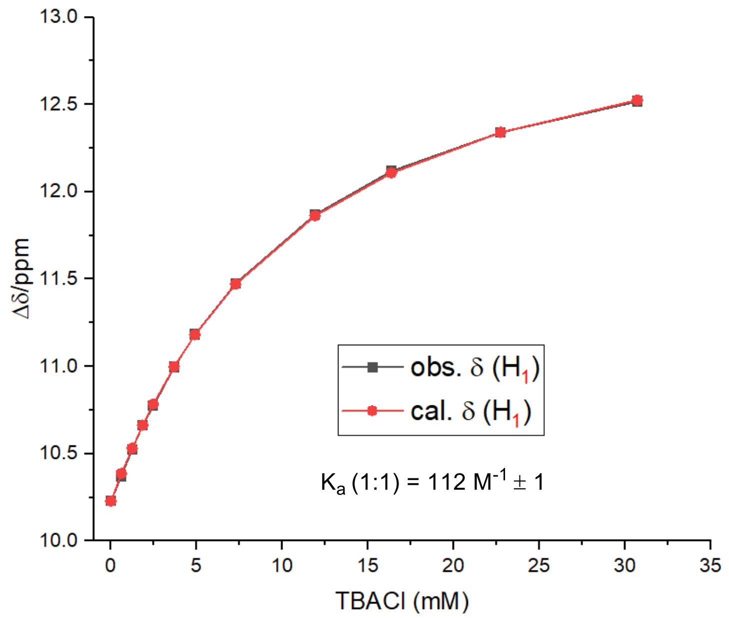

Figure S42. The plot of chemical shift (δ) of H_1 proton *vs* concentration of TBACl added, fitted to 1:1 binding model of BindFit v0.5.

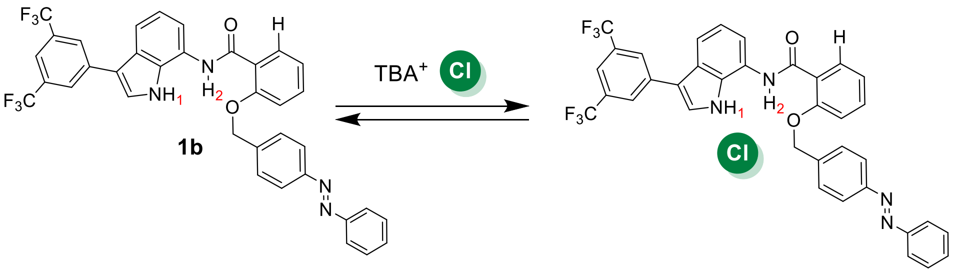

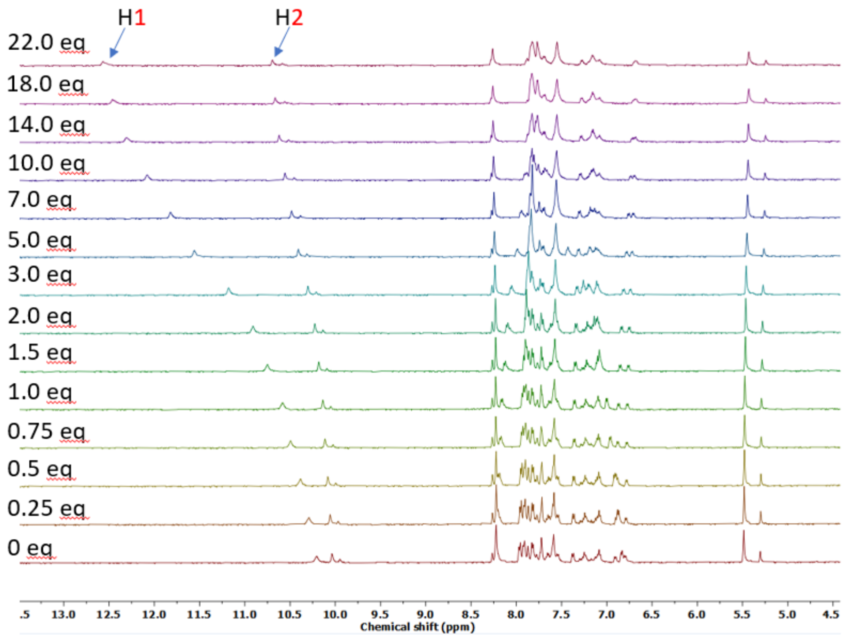

Figure S43. ^{1}H NMR titration spectra for **1b** (2.5 mM in CD_3CN) with stepwise addition of TBACl in CD_3CN. The equivalents of added TBACl are shown on the stacked spectra.

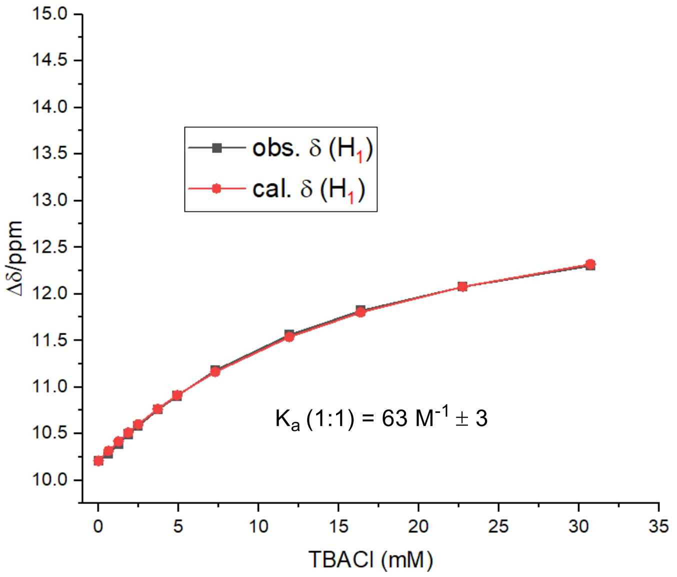

Figure S44. The plot of chemical shift (δ) of H_1 proton *vs* concentration of TBACl added, fitted to 1:1 binding model of BindFit v0.5.

III. Ion transport studies

Vesicle preparation

A thin film of lipid (1-palmitoyl-2-oleoyl-*sn*-3-phosphatidylcholine POPC or dipalmitoyl phosphatidylcholine DPPC) was formed by evaporating a chloroform solution under a stream of nitrogen gas, and then under high vacuum for 6 hours. The lipid film was hydrated by vortexing with the prepared buffer (100 mM NaCl, 10 mM HEPES, 1 mM 8-Hydroxypyrene-1,3,6-trisulfonic acid trisodium salt (HPTS), pH 7.0). The lipid suspension was then subjected to 5 freeze-thaw cycles using liquid nitrogen and a water bath (40°C), followed by extrusion 19 times through a polycarbonate membrane (pore size 200 nm) at rt. Extrusion was performed at 50°C in the case of DPPC lipids. Extra-vesicular components were removed by size exclusion chromatography on a Sephadex G-25 column with 100 mM NaCl, 10 mM HEPES, pH 7.0. Final conditions: LUVs (2.5 mM lipid); inside 100 mM NaCl, 10 mM HEPES, 1 mM HPTS, pH 7.0; outside: 100 mM NaCl, 10 mM HEPES, pH 7.0.

Transport assays with HPTS

In a typical experiment, the LUVs containing HPTS (40 µL, final lipid concentration 31.3 µM) were added to buffer (2910 µL of 100 mM NaCl, 10 mM HEPES, pH 7.0) at 25°C under gentle stirring. A pulse of NaOH (30 µL, 0.5 M) was added at 20 s to initiate the experiment. At 100 s the test transporter was added, followed by detergent (40 µL of Triton X-100 in 7:1 (v/v) H_2O-DMSO) at 300 secs to calibrate the assay. The fluorescence emission was monitored at λ_{em} = 510 nm (λ_{ex} = 460/405 nm). The fractional fluorescence intensity (I_{rel}) was calculated from equation (S1), where R_t is the fluorescence ratio at time t, (ratio of intensities 460 nm / 405 nm excitation) R_0 is the fluorescence ratio at time 77 s, and R_d is the fluorescence ratio after the addition of detergent.

$$I_{rel} = \frac{R_t - R_0}{R_d - R_0} \qquad \textbf{(S1)}$$

The fractional fluorescence intensity (I_{rel}) at 290 s just prior to lysis, defined as the fractional activity y, was plotted as a function of the ionophore concentration (x / µM). Hill coefficients (n) and EC_{50} values were calculated by fitting to the Hill equation (S2) equation,

$$y = y_0 + (y_{max} - y_0).\frac{x^n}{EC50^n + x^n} \qquad \textbf{(S2)}$$

where y_0 is the fractional activity in the absence of transporter, y_{max} is the fractional activity in with excess transporter, x is the transporter concentration in the cuvette.

For all compounds each individual concentration was repeated at-least three times and averaged; error bars represent standard deviations.

Experiments with DPPC lipids were conducted in the same way. For elevated temperature studies, the sample was equilibrated at 45°C (using the Peltier temperature controller) for 5 minutes prior to initiating the experiment.

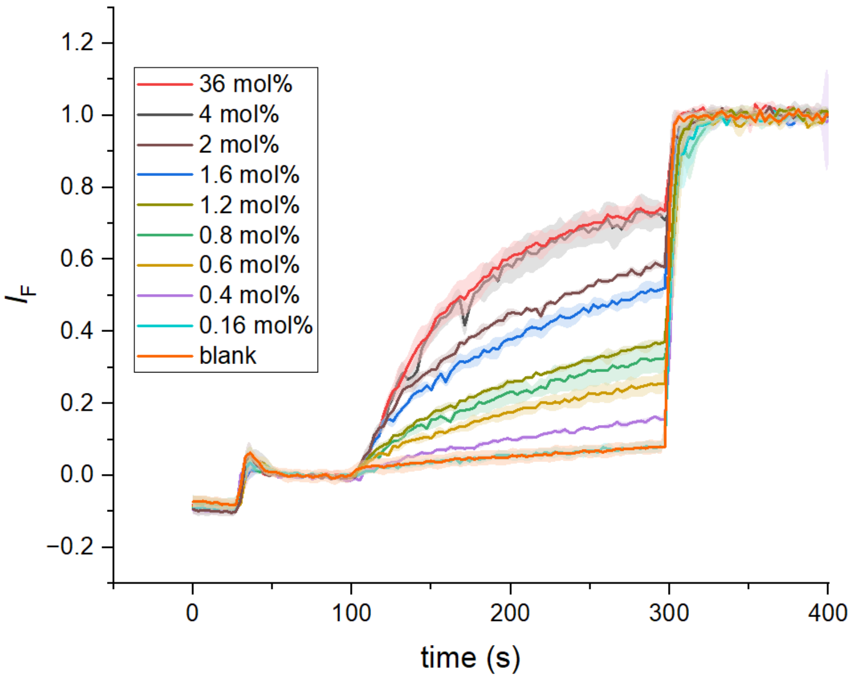

Figure S45. Ion transport HPTS assay data for **1** in POPC LUVs.

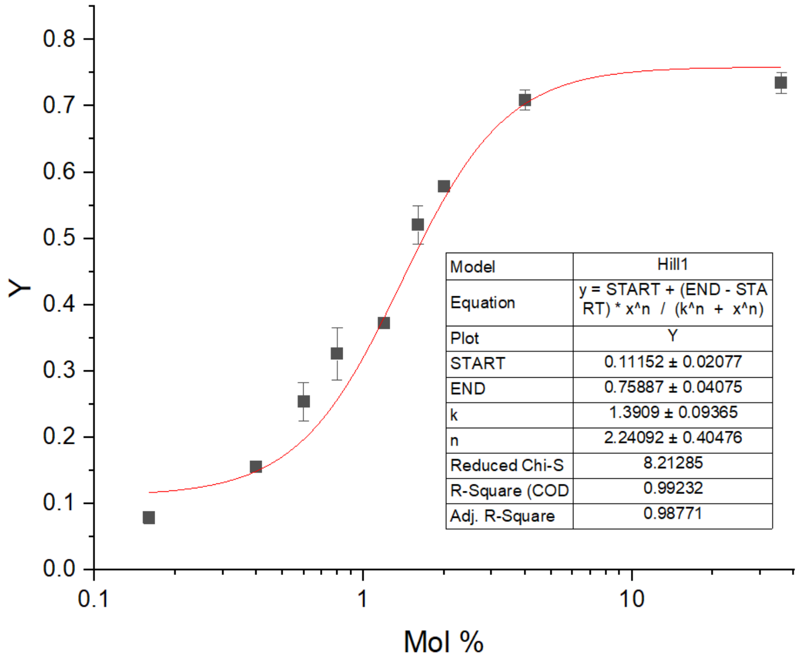

Figure S46. Hill plot for **1**.

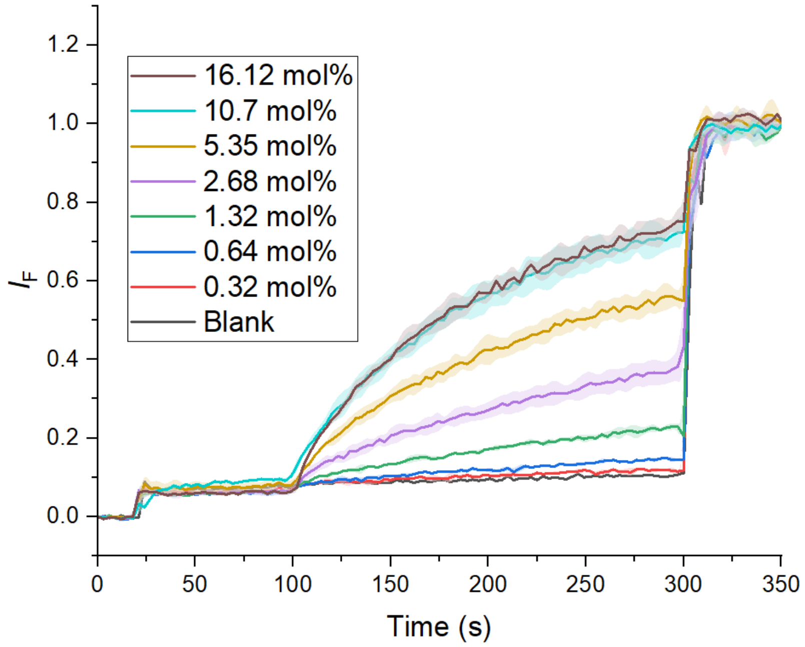

Figure S47. Ion transport HPTS assay data for **2** in POPC LUVs.

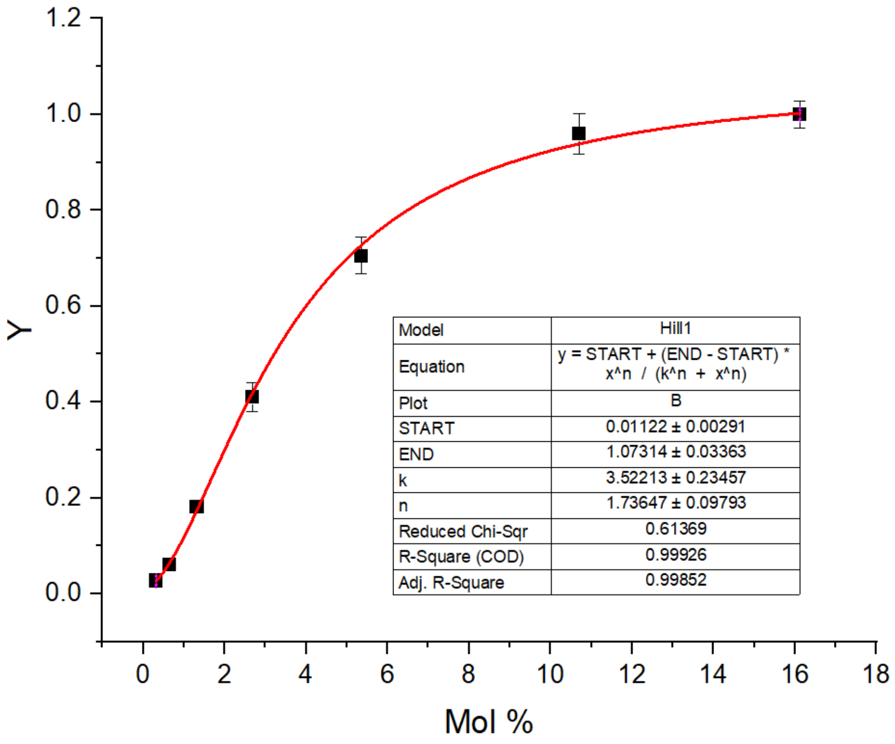

Figure S48. Hill plot for **2**.

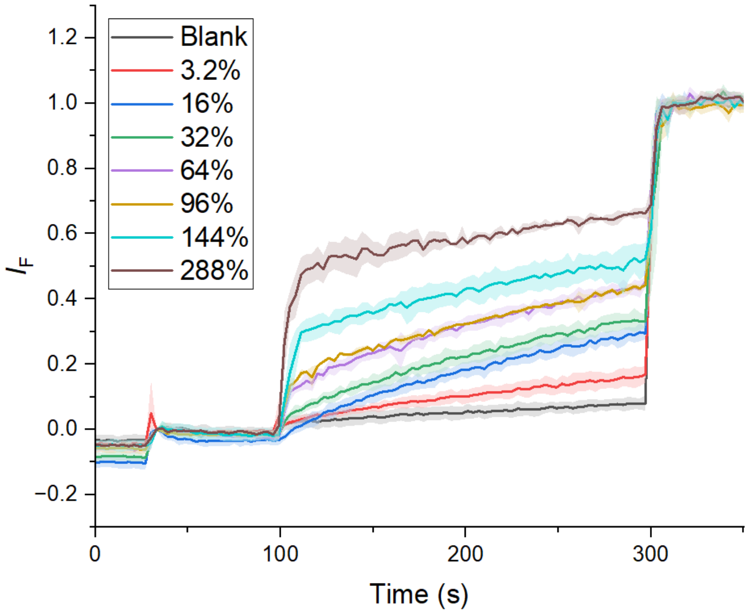

Figure S49. Ion transport HPTS assay data for **3** in POPC LUVs.

Effect of external ion exchange

The effect of external ion exchange was explored by using a previously reported external ion exchange assay.[5-7] These experiments were carried out by adding the POPC vesicle solution (prepared as above) to buffer (100 mM MX, 10 mM HEPES, pH 7.0), where M = Li, Na, K, Rb, Cs (X = Cl), and X = Cl, Br, I, NO_3, and ClO_4 (M = Na).

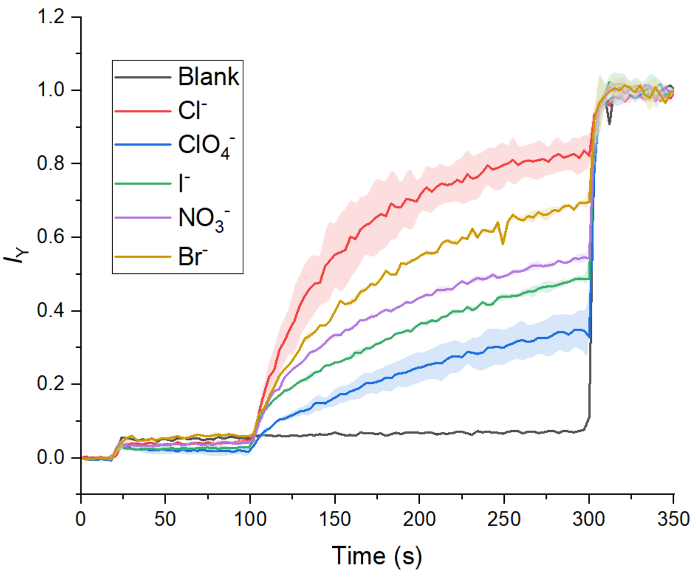

Figure S50. HPTS assay of **1** at 4 mol% carrier concentration and varying external anions (external buffer: 100 mM NaX (X = Cl, Br, I, NO_3, and ClO_4, 10 mM HEPES).

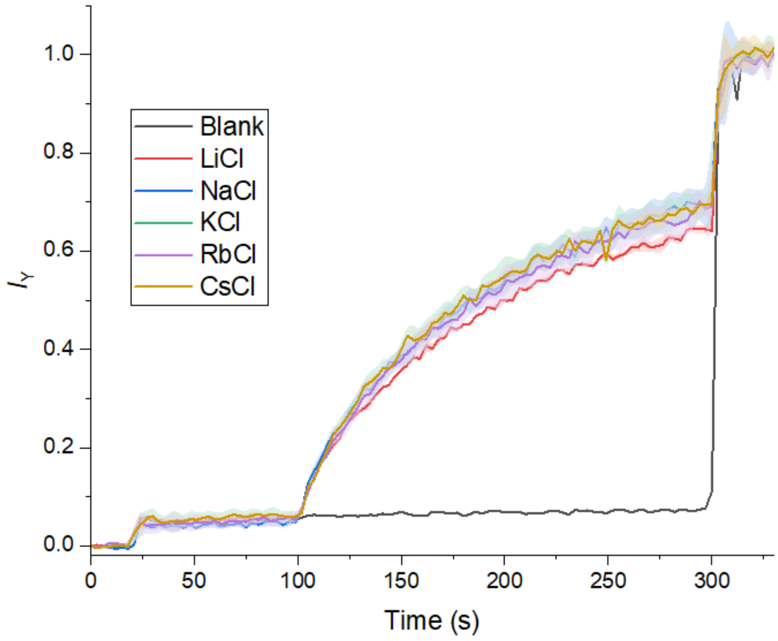

Figure S51. HPTS assay of **1** at 3 mol% carrier concentration and varying external anions (external buffer: 100 mM MCl (M = Li, Na, K, Rb, and Cs, 10 mM HEPES).

Lucigenin assay with external SO_4^{2-} and NO_3^- anions

Preparation of POPC-LUVs⊃lucigenin vesicles: In a 10 mL clean and dry round bottom flask, the thin transparent film of POPC was formed by drying 1.0 mL lipid (POPC, 25 mg/mL in $CHCl_3$) while providing continuous rotation and purging nitrogen. The transparent thin film was kept under a high vacuum for 4 hours to remove all traces of $CHCl_3$. Then the transparent thin film was hydrated with 1.0 mL aqueous NaCl (200 mM, 1.0 mM Lucigenin) buffered at pH of 6.5 with occasional vortexing at 10 min intervals for 1 h. The resulting suspension was subjected to freeze and thaw cycles (≥ 10 liquid nitrogen, 55 °C water bath) and 21 times extrusion through 200 nm pore size polycarbonate membrane. The size exclusion chromatography (using Sephadex G-50) was performed to remove extravesicular dye using 200 mM NaCl solution as eluent. The collected vesicles suspension was diluted to 4 mL. Final conditions: ~ 5 mM POPC; inside: 200 mM NaCl, 1 mM lucigenin, pH 6.5; outside: either 200 mM $NaNO_3$ or 200 mM Na_2SO_4.

Ion transport assay

In a clean and dry fluorescence cuvette, either 200 mM of $NaNO_3$ or 200 mM of Na_2SO_4 (2980 µL) and POPC-LUVs⊃lucigenin (20 µL, 54.8 µM) was added. This suspension was placed in a slowly stirring condition in a fluorescence instrument equipped with a magnetic stirrer (at t = 0 s). The fluorescence intensity of lucigenin was monitored at λ_{em} = 535 nm (λ_{ex} = 455 nm) as a course of time. The transporter molecule **1** (20 mol%) was added at t = 50 s. Finally, vesicles were lysed by adding 10% Triton X-100 (40 µL) at t = 150 s for the complete destruction of chloride gradient.

The time-dependent data were normalized to percent change in fluorescence intensity using Equation S3.

$$I_F = [(I_t - I_0) / (I_\infty - I_0)] \times (1) \qquad \text{Equation S3}$$

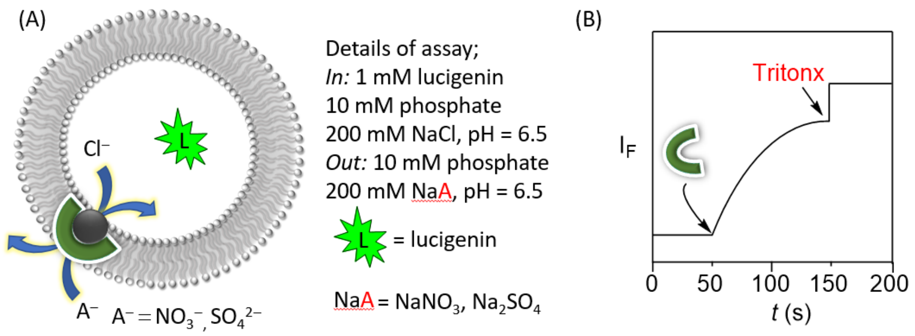

Figure S52. (A) Representation of fluorescence-based antiport assay using POPC-LUVs⊃lucigenin. (B) Representation of ion transport kinetics showing normalization window

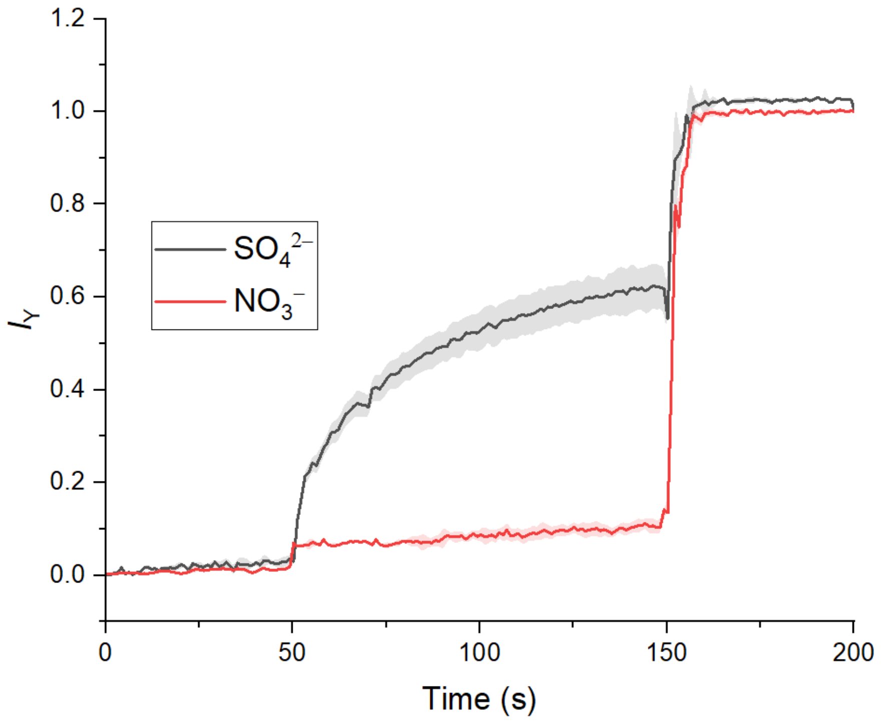

Figure S53. Ion transport activity of **1** (20 mol%) in presence of external SO_4^{2-} and NO_3^- ions.

IV. Stimulus-responsive triggered activation in the solution phase

Assessment of photolysis of protransporter 1a to give 1 upon irradiation at 405 nm: In a clean and dry NMR tube, the solution of **1a** was taken in DMSO-d_6 (1 mM in 0.5 mL). The ^{1}H NMR spectrum of the sample was recorded first (t = 0 min). Then, the NMR tube containing the compound **1a** was photoirradiated using 405 nm LED (1W) for different time intervals. The ^{1}H NMR spectrum of the irradiated samples were recorded at the end of each irradiation. All ^{1}H NMR spectra were processed using MestReNova 6.0 by considering the residual solvent peak as an internal reference. Finally, the NMR spectra of **1a** and the photoirradiated samples were stacked and compared with as synthesized **1** (recorded in DMSO-d_6). Upon photoirradiation, the appearance and disappearance of the different proton peak signals of the protransporter **1a** indicated the release of as-synthesized active transporter **1**.

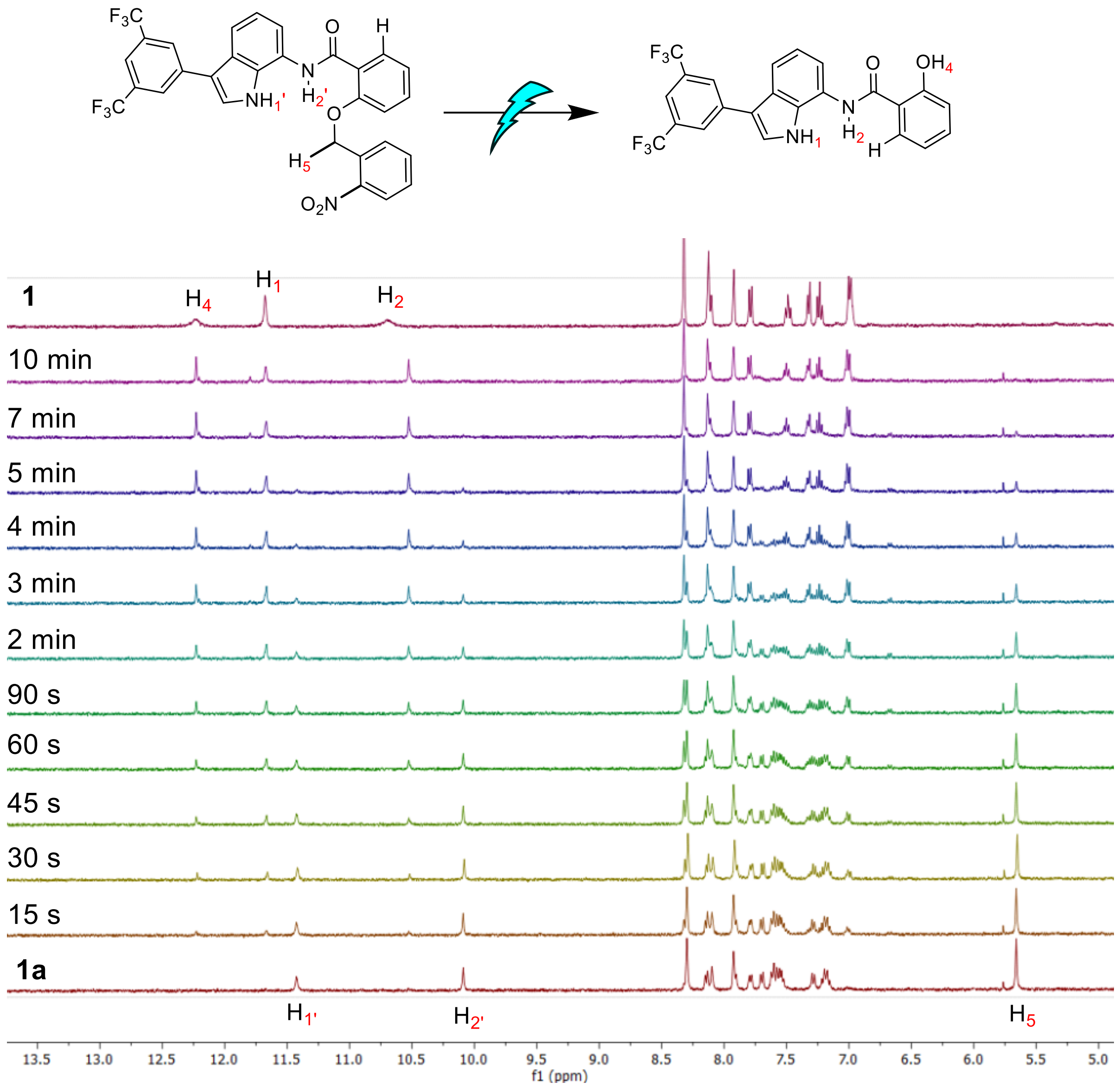

Figure S54. Photo release of active transporter **1** from protransporter **1a** upon photoirradiation using 405 nm LEDs (1 W) recorded in DMSO-d_6.

<u>Assessment of triggered release of 1 from 1b using ammonium formate:</u>

UV-Vis absorption studies:

1b (100 µM, 2:1 MeOH:THF solution) was taken in a 1 mL of cuvette and absorption spectra was recorded. Zn (10 eq) and aqeous ammonium formate (20-120 eq) were then added and absorption spectra was recorded at different time intervals while keeping the mixture at 37 ^{0}C using a Peltier temperature controller. The UV-vis absorption spectra showed the concomitant decrease in the absortion band at ~ 350 nm upon increasing the concentration of ammonium formate indication the reduction of azo functionality.

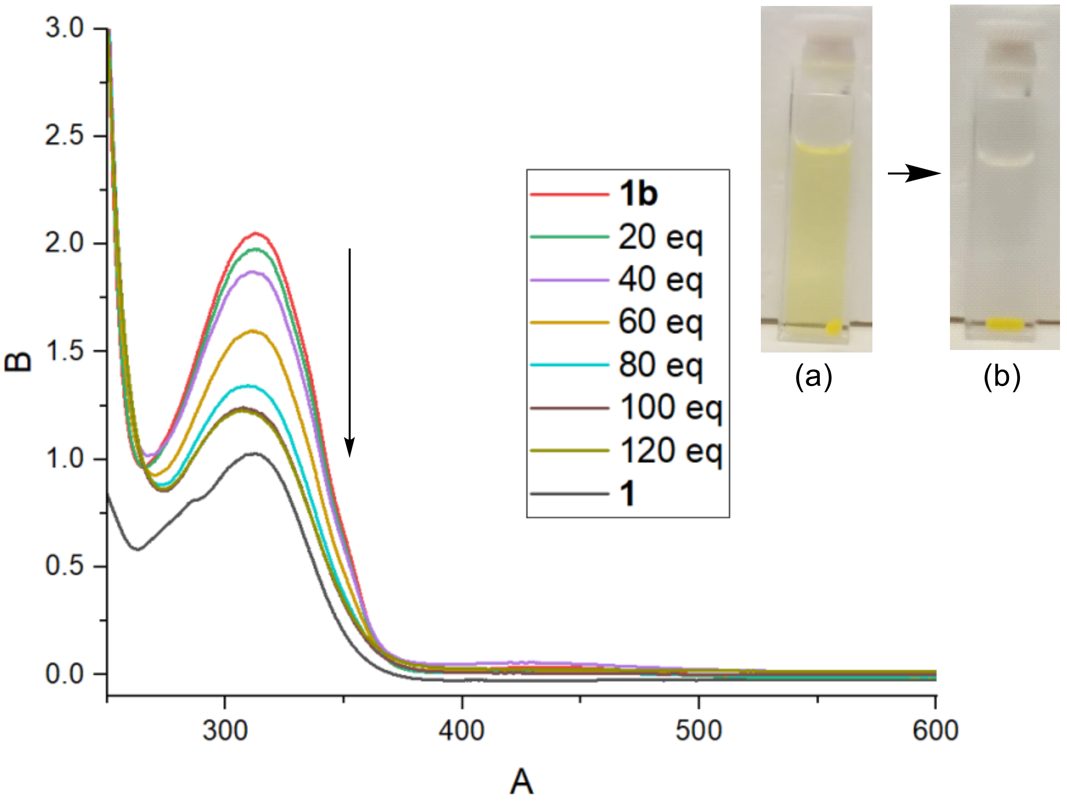

Figure S55. UV-vis spectra of **1b** in presence of ammonium formate and zinc. (a) Solution of **1b** in a mixture of MeOH:THF (1:1) (b) After addition of zinc (10 eq) and ammonium formate (100 eq). **Note:** colour changes from yellow to colourless solution indicates the reduction of the azo group.

HPLC analysis:

A stock solution of **1b** (0.5 mM, 2mL) was prepared in a mixture of THF and MeOH (1:2). To this solution, Zinc powder (10 eq.) was added. Subsequently, an aqueous solution of ammonium formate (50-200 eq) was added to the above mixture and after each addition, the mixture was stirred at 37 °C for 10 min. 200 µL of this mixture after each addition was filtered (Fisher band, PTFE 0.2 uM) and analysed using high-performance liquid chromatography (Thermo Scientific Vanquish Core HPLC: mobile phase was H_2O/CH_3CN; stationary phase C-18 reverse phase column (Ascentis, 5 µm, 15 cm x 4.6 mm)). CH_3CN concentration was increased from 5% to 95% over 10 min and maintained at 95% for another 5 min at a flow rate of 2 mL/min. The formation of active transporter **1** was monitored by a UV detector with excitation at 300 nm. Similarly, pure **1b** and **1** were also subjected to HPLC analysis. The traces of pure **1**, caged **1b**, and decaged samples were stacked in Origin2023 (64 bit). The retention time for active transporter **1** and caged protransporter **1b** were found to be 13.03 min and 10.56 min, respectively.

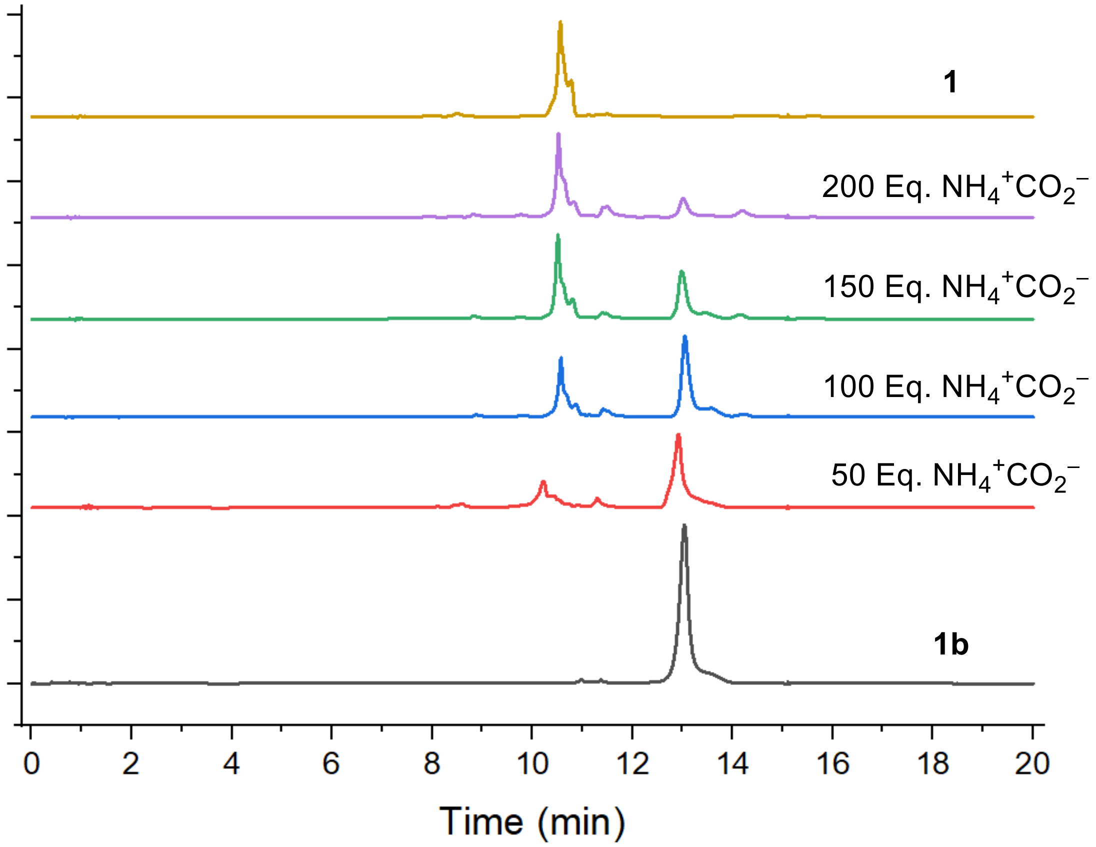

Figure S56. HPLC traces showing the release of **1** by treating **1b** with ammonium formate and zinc.

V. Stimulus-responsive ion transport activation

Activation of 1a with light in HPTS-based vesicles:

Preparation of POPC-LUVs⊃HPTS vesicles: These vesicles were prepared using the procedure as described above.

Experiment: In a clean and dry fluorescence cuvette, LUVs containing HPTS (40 µL, final lipid concentration 31.3 µM), and and **1a** (4.0 mol%) were taken. A pulse of NaOH (30 µL, 0.5 M) was added at 20 s to generate the pH gradient across the membrane to initiate the ion transport and finally detergent (40 µL of Triton X-100 in 7:1 (v/v) H_2O-DMSO) at 300 secs to calibrate the assay. Negligible transport activity was observed for **1a**. Samples containing HPTS-bound vesicles (40 µL) and **1a** (4.0 mol%) were initially photoirradiated at 405 nm of light using LEDs (1 W) for different time intervals and ion transport was monitored after each photoirradiation process. A significant enhancement in the ion transport activity was oberved upon photoirradiation at 405 nm of light, indicative of the formation of active transporter **1**.

The time-dependent data were normalized to percent change in fluorescence intensity using Equation S1.

Activation of 1b with redox in Lucigenin-based vesicles:

Preparation of POPC-LUVs⊃lucigenin vesicles: In 10 mL clean and dry round bottom flask, the thin transparent film of POPC was formed by drying 1.0 mL lipid (POPC, 25 mg/mL in $CHCl_3$) whilst providing continuous rotation and purging nitrogen. The transparent thin film was kept under a high vacuum for 4 hours to remove all traces of $CHCl_3$, before it was hydrated with 1.0 mL aqueous $NaNO_3$ (200 mM, 1.0 mM Lucigenin) with pH of 6.5 using 10 mM phosphate buffer with occasional vortexing at 10 min intervals for 1 h. The resulting suspension was subjected to freeze and thaw cycles (≥ 10 liquid nitrogen, 55 °C water bath) and 21 times extrusion through 200 nm pore size polycarbonate membrane. Size exclusion chromatography (using Sephadex G-50) was performed to remove extravesicular dye using 200 mM $NaNO_3$ solution as eluent. The collected vesicles suspension was diluted to 4 mL. Final conditions: ~ 5 mM POPC; inside: 200 mM $NaNO_3$, 1 mM lucigenin, pH = 6.5; outside: 200 mM $NaNO_3$, pH = 6.5.

Ion transport activity across Lucigenin-based vesicles:

Before performing the activation of **1b**, dose-dependent transport activity of **1** was performed to find out an optimum concentration needed for the activation process (Figure S74). To perform the experiment, in clean and dry fluorescence cuvette, 200 mM $NaNO_3$ (2910 μL), POPC-LUVs⊃lucigenin (20 μL, 54.8 μM) and ion transporter **1** (varying conc., 20 μL from THF solution) were added. This suspension was placed in a slowly stirring condition in a fluorescence instrument equipped with a magnetic stirrer (at $t = 0$ s). The fluorescence intensity of lucigenin was monitored at λ_{em} = 535 nm (λ_{ex} = 455 nm) over time. The chloride gradient was created by the addition of 2.0 M NaCl (50 μL) at $t = 50$ s between intra- and extravesicular solutions. Finally, vesicles were lysed by adding 10% Triton X-100 (40 μL) at $t = 150$ s for the complete destruction of the chloride gradient.

The time-dependent data were normalized to percent change in fluorescence intensity using Equation S4:

$$I_F = [(I_t - I_0) / (I_\infty - I_0)] \times (-1) \qquad \text{Equation S4}$$

where, I_0 is the initial intensity, I_t is the intensity at time t, and I_∞ is the final intensity after addition of Triton X-100.

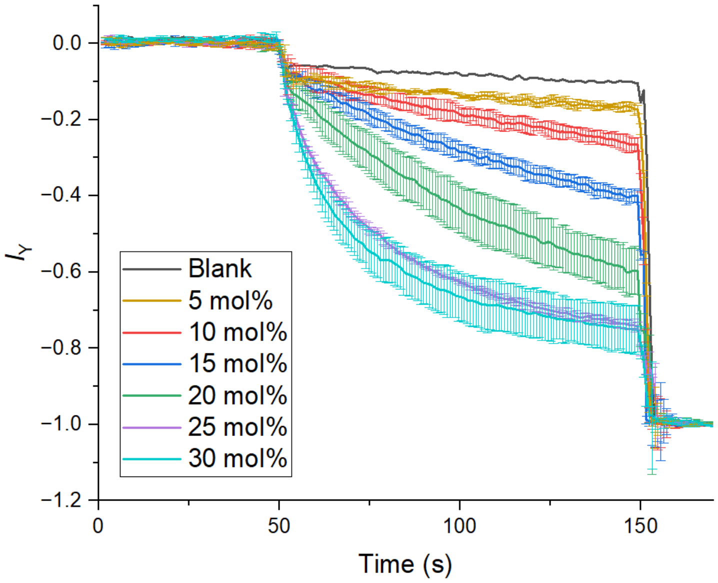

Figure 57: Transport activity of **1** across lucigenin-based vesicles.

Activation with ammonium formate: In a clean and dry fluorescence cuvette, 200 mM of $NaNO_3$ (2910 µL), POPC-LUVs⊐lucigenin (20 µL, 54.8 µM), and **1b** (30 mol%) were taken. The fluorescence intensity of lucigenin was monitored at λ_{em} = 535 nm (λ_{ex} = 455 nm) as a course of time by creating a chloride gradiant across the membrane by adding NaCl. Negligible transport activity was observed for **1b**. Subsequently, in a separate cuvette, zinc (10.0 eq) and aqueous ammonium formate (25-200 eq) were added to **1b** (2.5 mM) in a mixture of MeOH:THF (2:1) and stirred at 37 ^{0}C. 20 µL (16.6 µM, 30 mol%) of this solution after each addition of ammonium formate was added to the fluorescence cuvette containing 200 mM of $NaNO_3$ (2910 µL) and lucigenin-based vesicles (20 µL, 54.8 µM) and ion transport was monitored each time. Significant enhancement in the ion transport indicates the reduction of azobenzene subunit to trigger the formation of active transporter **1**.

The time-dependent data were normalized to percent change in fluorescence intensity using Equation S4.

VI. References

(1) http://app.supramolecular.org/bindfit/.
